# Dual functionality of *cis*-regulatory elements as developmental enhancers and Polycomb response elements

**DOI:** 10.1101/gad.292870.116

**Published:** 2017-03-15

**Authors:** Jelena Erceg, Tibor Pakozdi, Raquel Marco-Ferreres, Yad Ghavi-Helm, Charles Girardot, Adrian P. Bracken, Eileen E.M. Furlong

**Affiliations:** 1 Genome Biology Unit, European Molecular Biology Laboratory (EMBL), Heidelberg D69117, Germany;; 2 Smurfit Institute of Genetics, Trinity College Dublin, Dublin 2, Ireland

**Keywords:** Polycomb response elements (PREs), pleiohomeotic repressive complex (PhoRC), developmental enhancers, spatio–temporal expression, transcriptional repression, silencing, embryonic development

## Abstract

Developmental gene expression is tightly regulated through enhancer elements, which initiate dynamic spatio–temporal expression, and Polycomb response elements (PREs), which maintain stable gene silencing. These two *cis*-regulatory functions are thought to operate through distinct dedicated elements. By examining the occupancy of the *Drosophila* pleiohomeotic repressive complex (PhoRC) during embryogenesis, we revealed extensive co-occupancy at developmental enhancers. Using an established in vivo assay for PRE activity, we demonstrated that a subset of characterized developmental enhancers can function as PREs, silencing transcription in a Polycomb-dependent manner. Conversely, some classic *Drosophila* PREs can function as developmental enhancers in vivo, activating spatio–temporal expression. This study therefore uncovers elements with dual function: activating transcription in some cells (enhancers) while stably maintaining transcriptional silencing in others (PREs). Given that enhancers initiate spatio–temporal gene expression, reuse of the same elements by the Polycomb group (PcG) system may help fine-tune gene expression and ensure the timely maintenance of cell identities.

## Abstract (precis)

Here, Erceg et al. studied the occupancy of the *Drosophila* PhoRC during embryogenesis and revealed extensive co-occupancy at developmental enhancers. By using an established in vivo assay for Polycomb response element (PRE) activity, they show that a subset of characterized developmental enhancers can function as PREs and silence transcription in a Polycomb-dependent manner, thereby suggesting that reuse of the same elements by the PcG system may help fine-tune gene expression and ensure the timely maintenance of cell identities.

Polycomb group (PcG) proteins are an evolutionarily conserved chromatin-modifying system that functions to maintain gene silencing during development, having an essential role in lineage commitment and differentiation (Schwartz and Pirrotta 2013; Simon and Kingston 2013; Geisler and Paro 2015; Piunti and Shilatifard 2016). The system was first identified in *Drosophila*, where PcG loss-of-function mutations cause derepression of *Hox* genes in body segments where they are normally not expressed, leading to dramatic changes in segment identity (Lewis 1978; Struhl 1981; Duncan 1982). Biochemically, PcG proteins form several multiprotein complexes, including Polycomb-repressive complex 1 (PRC1) and PRC2 (for review, see Muller and Verrijzer 2009; Simon and Kingston 2009; Beisel and Paro 2011). PRC2 contains an enzyme that methylates Lys27 of H3 to generate H3K27me3 (Czermin et al. 2002; Muller et al. 2002), a chromatin modification essential for PRC2-mediated silencing (Pengelly et al. 2013), while PRC1 contains proteins that recognize H3K27me3, which may help direct its recruitment (Czermin et al. 2002). H3K27me3 often spreads across large domains (Schuettengruber et al. 2009) such that a gene's entire regulatory landscape (including the promoter, gene body, and enhancers) may be part of a repressed three-dimensional PcG state (Bantignies and Cavalli 2011). This stable repression is antagonized by the Trithorax (Trx) group proteins, which function as anti-repressors to counteract PcG function (Klymenko and Muller 2004).

How PcG proteins are targeted to specific genomic locations remains a topic of active debate (Muller and Kassis 2006; Bauer et al. 2016). Although almost all components of the PcG system are maternally deposited and ubiquitously expressed, at least in *Drosophila*, they target only a subset of genes. In *Drosophila*, PcG proteins are recruited to chromatin via Polycomb response elements (PREs), *cis*-regulatory elements that silence transcription in a PcG-dependent manner (Simon et al. 1993). Pho, which, together with dSfmbt, forms the pleiohomeotic repressive complex (PhoRC) (Klymenko et al. 2006), binds to PREs in a sequence-specific manner (Brown et al. 1998; Fritsch et al. 1999). PhoRC directly interacts with components of PRC1 (Frey et al. 2016) and PRC2 (Wang et al. 2004) and is thus thought to recruit these complexes to specific regions of the genome. PREs are operationally defined as genomic elements capable of mediating PcG-dependent transcriptional silencing of associated target genes; for instance, in transgenic reporter assays (Kassis and Brown 2013). There are ∼30 operationally defined *Drosophila* PREs to date (Supplemental Table S1), which appear to act in a dominant manner to silence transcription of any linked gene (Sengupta et al. 2004). While some PREs are located several kilobases away from the silenced gene's promoter (e.g., in the *Hox* loci), most non-Hox target PREs are close to the transcriptional start site (TSS) (Supplemental Fig. 2; Oktaba et al. 2008). Recent genome-wide studies in whole embryos and tissue culture cells have identified thousands of regions bound by Pho and/or components of PRC1 or PRC2 (Negre et al. 2006; Schwartz et al. 2006; Tolhuis et al. 2006; Kwong et al. 2008; Schuettengruber et al. 2009, 2014; Ray et al. 2016), suggesting that there are hundreds if not thousands of PREs throughout the *Drosophila* genome. However, the functional requirement and general properties of these elements remain poorly characterized.

In addition to *cis*-regulatory elements dedicated to gene silencing, the activation of gene expression is regulated through enhancer elements, *cis*-regulatory elements that recruit multiple transcription factors (TFs) to activate specific patterns of spatio–temporal expression (Spitz and Furlong 2012). *Drosophila* has served as an excellent model system to study enhancer activity in vivo; the spatial and temporal activity of ∼5000 enhancers has been characterized during *Drosophila* embryogenesis to date (Vienna tiles [Kvon et al. 2014], RedFly [Gallo et al. 2011], and CAD [Bonn et al. 2012a]), providing a rich resource of regulatory elements that activate transcription in a huge diversity of cell types and developmental stages. While enhancers act as the key drivers to initiate very dynamic temporal and spatial gene expression, the PcG system helps to maintain these expression states through stable silencing in cells where the gene should not be expressed (Schwartz and Pirrotta 2013; Simon and Kingston 2013; Geisler and Paro 2015; Piunti and Shilatifard 2016). Both types of regulatory elements—enhancers and PREs—are assumed to act as separate dedicated elements, recruiting different sets of TFs and associated complexes.

To better understand the relationship between PREs and enhancer elements, we performed an in-depth analysis of the functional properties of *cis*-regulatory elements during embryonic development. To initiate this study, we performed ChIP-seq (chromatin immunoprecipitation [ChIP] combined with high-throughput sequencing) of both components of the PhoRC (Pho and dSfmbt) during a narrow 2-h time window of embryogenesis when major cell lineages are specified within the mesoderm and ectoderm. This identified almost 1000 regions cobound by both proteins, a surprising fraction of which is bound to characterized developmental enhancers. To determine whether Polycomb can mediate silencing through these elements, we investigated whether PhoRC-bound enhancers can act as PREs in vivo. Using two established functional assays for PRE activity, we demonstrated that 50% of enhancers tested can function as PREs in vivo, silencing transcription in a PcG-dependent manner. Conversely, we show that a subset of characterized “classic” PREs can function as developmental enhancers in vivo, activating transcription in specific spatial domains. Therefore, in addition to dedicated enhancers and dedicated PREs, this study identified *cis*-regulatory elements with dual activity, functioning as developmental enhancers to activate spatio–temporal expression in one cell type and PREs that stably silence transcription in another. Having both functions mediated through the same element may provide more fine-tuning of gene expression and ensure that key enhancers are rapidly and stably silenced during key lineage transitions.

## Results

### PhoRC binds to developmental enhancers during embryogenesis

**Figure 1.**
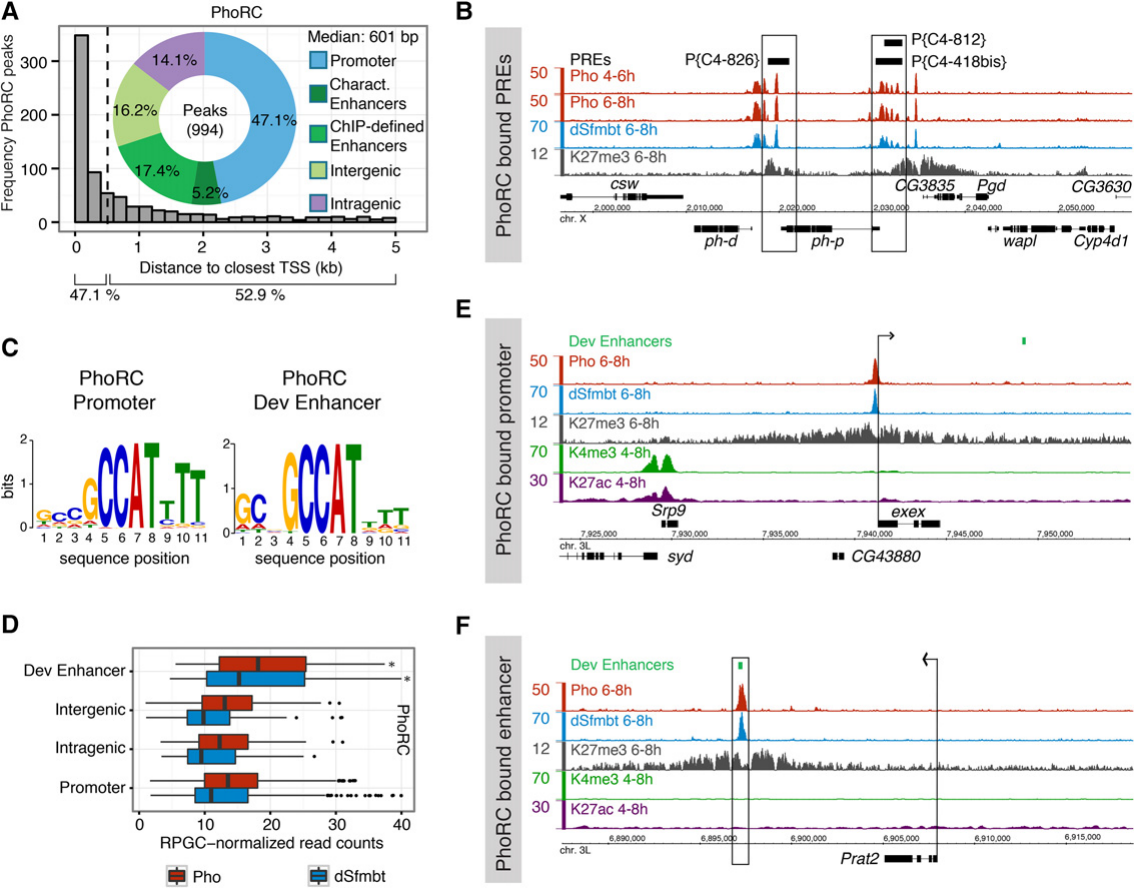
Distribution of PhoRC binding at developmental enhancers. (*A*) Frequency of PhoRC ChIP peaks relative to the distance from the closest TSS (histogram). Percentage of PhoRC peaks (doughnut) overlapping five genomic categories; 22.6% of PhoRC peaks are within developmental enhancers (5.2% characterized in transgenic embryos; 17.4% TF-ChIP-defined enhancers). (*B*) Mesodermal Pho (red) and dSfmbt (blue) binding (input-subtracted ChIP signal) at characterized PREs (black boxes) (Bloyer et al. 2003) with PRC2-associated H3K27me3 (gray; H3 subtracted) (Bonn et al. 2012a). (*C*) De novo discovered Pho motif at PhoRC-bound promoters and developmental enhancers. (*D*) Quantitative ChIP signal (read counts) for Pho (red) and dSfmbt (blue) at 6–8 h at enhancer-bound, intergenic-bound, intragenic-bound, and promoter-bound regions. The PhoRC ChIP signal is significantly higher at enhancers versus promoters. *P*-value = 1.62 × 10^−10^ for Pho; *P*-value = 7.71 × 10^−10^ for dSfmbt, Mann-Whitney two-sided *U*-test. (*E*,*F*) Mesodermal Pho binding (red), dSfmbt binding (blue) (input-subtracted ChIP signal), and H3K27me3, (gray; H3 subtracted) (Bonn et al. 2012a) and whole-embryo H3K4me3 (green) and H3K27ac (purple; H3 subtracted, from modENCODE). Examples illustrate the PhoRC-bound promoter (*E*) and PhoRC-bound enhancer (green box) (*F*). Arrows point to the direction of the gene's transcription.

To dissect the role of PhoRC in the regulation of cell type-specific developmental programs, we obtained a high-resolution map of Pho and dSfmbt occupancy specifically in mesodermal cells using BiTS-ChIP-seq (batch isolation of tissue-specific chromatin for immunoprecipitation [BiTS-ChIP] combined with high-throughput sequencing) (Bonn et al. 2012a) during two consecutive 2-h windows of embryogenesis. These time points span stages when mesodermal cells are multipotent (4–6 h) and are specified into mesodermal sublineages (6–8 h) and represent a more refined spatio–temporal resolution than previously examined in whole embryos (Kwong et al. 2008; Oktaba et al. 2008; Schuettengruber et al. 2009, 2014). We identified 1248 and 2460 high-confidence peaks for Pho and dSfmbt (6–8 h), respectively (combining peaks from 4–6 h and 6–8 h), 994 of which are co-occupied by both proteins (Supplemental Table S2), representing 79.6% of Pho-bound regions and 40% of dSfmbt-bound regions (Fig. 1A; Supplemental Fig. S1a,b). These cobound regions, referred to as PhoRC-bound regions, have quantitatively higher levels of both Pho and dSfmbt ChIP signal compared with regions bound by either protein alone (Supplemental Fig. S1c), suggesting that they bind with higher affinity as a complex to target sites. There is also a difference in the distribution of regions bound by PhoRC compared with regions bound by either protein alone (Supplemental Fig. S1): The majority (72.3%) of dSfmbt-only peaks is located very close to the promoter (97-base-pair [bp] median distance to the closest TSS), while Pho-only peaks are more loosely distributed around promoters (1890-bp median distance from the closest TSS) (Supplemental Fig. S1a,b), as observed in whole embryos (Kwong et al. 2008; Oktaba et al. 2008; Schuettengruber et al. 2009). The 994 PhoRC peaks have a distribution intermediate between that of dSfmbt and Pho alone (Fig. 1A, histogram; Supplemental Fig. S1d).

PhoRC-bound regions overlap 93% (28 out of 30) of all functionally characterized *Drosophila* PREs, including those at *hox* loci (Fig. 1B; Supplemental Fig. S2), and contain the known Pho motif (Fig. 1C; Oktaba et al. 2008; Schuettengruber et al. 2009), demonstrating the quality and sensitivity of the data. In many cases, our data further refine the boundaries of the characterized PREs (Supplemental Fig. S2) and identify many more putative PREs within these loci; e.g., *abd-A* (Supplemental Fig. S2d), *cad* (Supplemental Fig. S2k) and *Sox21b* (Supplemental Fig. S2n). The only exceptions are the *prod* and α*-PKC* loci (Supplemental Fig. S2p,q), which show no evidence of Polycomb-mediated repression in mesodermal cells at these developmental stages.

Although PhoRC often binds in close proximity to gene promoters (47% within 500 bp), more than half of the PhoRC peaks are found at greater distances (Fig. 1A, histogram). To examine where these non-TSS peaks reside, we categorized PhoRC peaks into five distinct genomic regions: (1) promoter TSSs (within 500 bp); (2) characterized developmental enhancers defined by a large collection of characterized enhancers tested in transgenic embryos (Gallo et al. 2011; Bonn et al. 2012a; Kvon et al. 2014); (3) ChIP-defined putative enhancers that we identified previously by TF occupancy (Zinzen et al. 2009; Junion et al. 2012) (importantly, we collectively tested >100 of these in transgenic embryos, and >95% function as developmental enhancers in vivo [Zinzen et al. 2009; Junion et al. 2012; Ciglar et al. 2014; Cannavo et al. 2015]); (4) intergenic regions, excluding enhancers; and (5) intragenic regions, including introns and exons.

In addition to binding close to TSSs, 225 of the 994 PhoRC sites reside within developmental enhancers, characterized either from transgenic embryos (52 elements, 5.2%) or within ChIP-defined putative enhancers (173 elements, 17.4%), representing 22.6% of all bound regions (Fig. 1A, Supplemental Table S3). This fraction represents a significant enrichment over matched background regions (4.85 log_2_ odds ratio; *P*-value = 8.53 × 10^−60^; Fisher's exact test) (Supplemental Fig. S3) and is likely an underestimate, since an additional 16.2% of PhoRC peaks reside within intergenic regions (Fig. 1A) containing H3K4me1 signal (Supplemental Fig. S4) and may represent as yet unidentified enhancer elements.

PhoRC occupancy at developmental enhancers represents prominent peaks as opposed to a low-level signal that may represent spurious binding; the level of Pho and dSfmbt-ChIP signal is higher at enhancer elements compared with promoter-bound regions (*P*-value = 1.62 × 10^−10^ for Pho signals; *P*-value = 7.71 × 10^−10^ for dSfmbt signals, Mann-Whitney *U*-test, two-sided) (Fig. 1D). Moreover, de novo motif discovery identified the known Pho motif (Kwong et al. 2008; Oktaba et al. 2008; Schuettengruber et al. 2009) in enhancer-bound peaks as well as in promoter-bound peaks (Fig. 1C), indicating that these enhancers have the capacity to directly recruit Pho. Taken together, these data indicate that, in addition to characterized PREs and promoter-proximal regions (Fig. 1B,E; Oktaba et al. 2008), a significant fraction of PhoRC binding occurs at developmental enhancers, many of which are located at large distances from promoter sequences (Fig. 1F).

### PhoRC is part of a functional PcG-repressive system at enhancers

**Figure 2.**
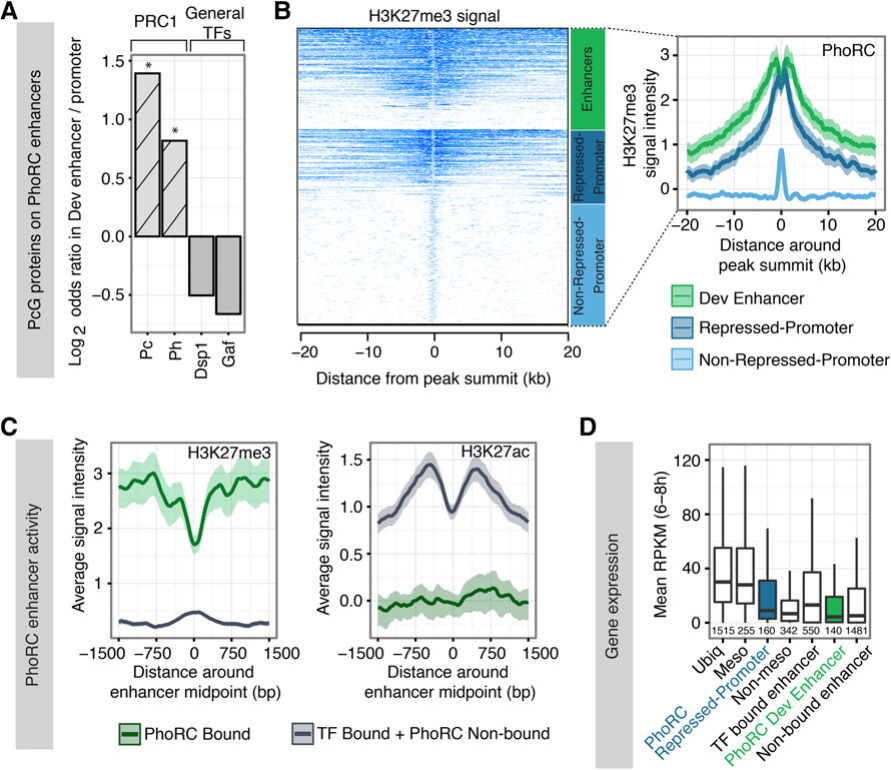
Developmental enhancers are bound by PcG proteins and associated with gene repression. (*A*) Pc and Ph (PRC1) occupancy is significantly higher at Pho-RC-bound developmental enhancers compared with PhoRC-bound promoter regions. Log_2_ odds ratios were 1.39 (Pc) and 0.79 (Ph). *P*-value = 3.24 × 10^−06^; 4*P*-value = .43 × 10^−03^, Fisher's exact test. The general TFs Dsp1 and Gaf are more enriched at PhoRC-bound promoters, although not significantly. (*) *P*-value < 0.05, Fisher's exact test. (*B*, *left*) Heat map showing H3K27me3 signal (H3 subtracted) centered on PhoRC peaks showing a bimodal broad distribution at enhancers (green) and repressed promoters (dark blue) and a unimodal peak at nonrepressed promoters (light blue). (*Right*) Average signal for each class; shadings indicate 95% confidence intervals from bootstrap estimation. (*C*) Enhancer signals for H3K27me3 and H3K27ac on PhoRC-bound (green) and PhoRC-nonbound (gray) enhancers, the latter being bound by developmental TFs. The shaded area indicates 95% confidence intervals. PhoRC-bound enhancers are enriched for H3K27me3 and depleted for H3K27ac, in contrast to TF-bound enhancers. (*D*) Mesoderm-specific gene expression at genes associated with PhoRC binding. The *Y*-axis shows the median RPKM (reads per kilobase per million reads) of mesoderm RNA sequencing (RNA-seq) data (Gaertner et al. 2012) on ubiquitous (ubiq), mesoderm-expressed (meso), and non-meso genes (Bonn et al. 2012a); genes associated with PhoRC-bound promoters (PhoRC-repressed promoter; dark blue) or enhancers (PhoRC dev enhancer; green); genes associated with TF-bound CRMs (TF-bound enhancer); and genes without occupancy of mesoderm-specific TFs at 6–8 h (nonbound enhancer). Genes with PcG-bound enhancers have low transcription, with RPKM levels similar to inactive genes.

The extensive occupancy of PhoRC at enhancers suggests that a proportion of Polycomb's activity is mediated through developmental enhancers and not only via spreading from previously characterized PREs and promoters (Fig. 1, cf. E and F). To assess whether PhoRC-bound enhancers can recruit a functional PcG system, we first determined whether other PcG proteins bind to enhancers. Available ChIP-seq data from whole embryos (Schuettengruber et al. 2009) indicate that two PRC1 components, Pc and Ph, are enriched at PhoRC-bound enhancers compared with PhoRC-bound promoters (Fig. 2A). Interestingly, the two general TFs Dorsal switch protein 1 (Dsp1) (Dejardin et al. 2005) and GAGA factor (Gaf; also known as Trx-like [Trl]) (Muller and Kassis 2006), suggested to aid in the recruitment of PcG proteins to PREs, are more depleted at PhoRC-bound enhancers compared with promoter-proximal regions (Fig. 2A). This suggests that our newly discovered PhoRC-bound enhancers may have different properties than promoter-proximal elements.

To evaluate PRC2 activity, we examined the levels and spread of the H3K27me3 signal at developmental enhancers compared with promoter-bound regions either bound or unbound by PhoRC using mesoderm-specific information on their chromatin state (Bonn et al. 2012a). Centering on the peak of PhoRC occupancy, the H3K27me3 signal at promoter-bound regions has two distributions—53.4% of promoters have a focused peak of H3K27me3 ± 500 bp of the PhoRC peak (Fig. 2B, bottom), which most likely represents PhoRC binding to stalled promoters (Enderle et al. 2011) and not classic Polycomb silencing. The remaining 46.6% of promoters have an approximately three times higher level of H3K27me3 signal, which spreads to approximately ±10 kb (Fig. 2B, middle). This broad H3K27me3-enriched promoter class includes all known Polycomb-repressed genes (e.g., the *Antp* and *Bithorax* hox loci) and is referred to here as “repressed promoters.” The bimodal distribution of H3K27me3 at repressed promoters (Schwartz et al. 2006) is in contrast to the unimodal peak at the nonrepressed promoters (Fig. 2B, right).

At PhoRC-bound enhancers, both the levels and spread of H3K27me3 are almost identical to that of PhoRC-bound repressed promoters (Fig. 2B, top). Moreover, PhoRC-bound enhancers have a nucleosome-depleted region (NDR) at the position of PhoRC binding (Fig. 2B), leading to a bimodal distribution of H3K27me3, similar to repressed promoters. This indicates that the quantitative levels and spread of PcG-mediated repression are similar regardless of whether it is emanating from a PhoRC-bound promoter-proximal element or a distal developmental enhancer (e.g., Fig. 1, E vs. F). To examine this further, we directly compared the activity state of enhancers bound by PhoRC with those bound by transcriptional activators (TFs) specifically in the mesoderm at these stages of development (Bonn et al. 2012a). PhoRC-bound-characterized enhancers are significantly enriched in the presence of H3K27me3 in mesodermal cells and depleted on PhoRC-nonbound enhancers (Fig. 2C). Conversely, the presence of H3K27ac, a mark associated with active enhancers (Creyghton et al. 2010; Rada-Iglesias et al. 2011; Bonn et al. 2012a), is depleted at PhoRC-bound enhancers in mesodermal cells while enriched at PhoRC-nonbound enhancers (Fig. 2C).

As H3K27me3 is genetically required for PRC2-mediated repression (Pengelly et al. 2013), these results suggest that PhoRC—and thereby H3K27me3—emanating from characterized enhancer elements could have a significant effect on transcriptional silencing, similar to H3K27me3 emanating from PcG-repressed promoters and characterized PREs. To assess this, we examined transcript levels of genes with PhoRC-bound promoters versus PhoRC-bound enhancers in their vicinity. Using mesoderm-specific RNA sequencing (RNA-seq) data (Gaertner et al. 2012), transcript levels for ubiquitous and mesoderm-specific genes were high, while genes not expressed in mesoderm were low (Fig. 2D), thereby serving as a reference for genes in an active and inactive state, respectively. The transcript levels of genes with broad H3K27me3 at their promoters are significantly reduced compared with that of active genes, as expected (PhoRC-repressed promoter) (Fig. 2D). Importantly, genes in the vicinity of PhoRC-bound-characterized enhancers (assigning to the nearest gene) appear strongly silenced, with transcript levels in the range of PhoRC-repressed promoters (Fig. 2D). In contrast, genes in the vicinity of characterized enhancers not bound by PhoRC but bound by mesoderm-specific TFs have significantly higher levels of expression (Fig. 2D).

These four lines of evidence—namely, the quantitative levels of H3K27me3 at PhoRC-bound enhancers, the spread of H3K27me3 from PhoRC-bound enhancers, the lack of H3K27ac at PhoRC-bound enhancers, and the transcript levels of the associated nearest genes—strongly suggest that PhoRC enhancer binding is associated with actively silenced enhancers, leading to the silencing of the associated genes’ expression.

### A subgroup of developmental enhancers functions as PREs in vivo

The extensive occupancy of PhoRC at previously characterized developmental enhancers is surprising and suggests that these elements may also function as PREs in vivo. In support of this, a PRE in the *eve* locus, which mediates PcG-mediated silencing in cells where *eve* is not expressed, also acts to maintain *eve* expression in the ventral nerve cord (Fujioka et al. 2008). Interestingly, although the mechanism is not understood, this activating function is also PcG-dependent. A PRE in the *en* locus was similarly shown to have positive effects on transcription in a manner independent of Trx genes (DeVido et al. 2008). However, other studies have concluded that PREs act solely to silence transcription (Simon et al. 1993), lacking enhancer-like properties to activate transcription (Kassis and Muller 2015).

To examine the functional properties of PhoRC-bound enhancers, we selected 16 regulatory elements, nine of which are characterized developmental enhancers, six of which are characterized PREs, and one, the *eve* element, that has been characterized as both. All elements contain a single DNase hypersensitivity site (DHS), with the exception of four regions that have no detectable DHS signal (using whole-embryo DHS data from Thomas et al. 2011). We reasoned that, depending on the cell state, the same regulatory element might act as a developmental enhancer in one context, recruiting tissue-specific TFs to initiate transcription, while acting as a PRE in another, recruiting PhoRC and PcG proteins to stably repress transcriptional activity.

**Figure 3.**
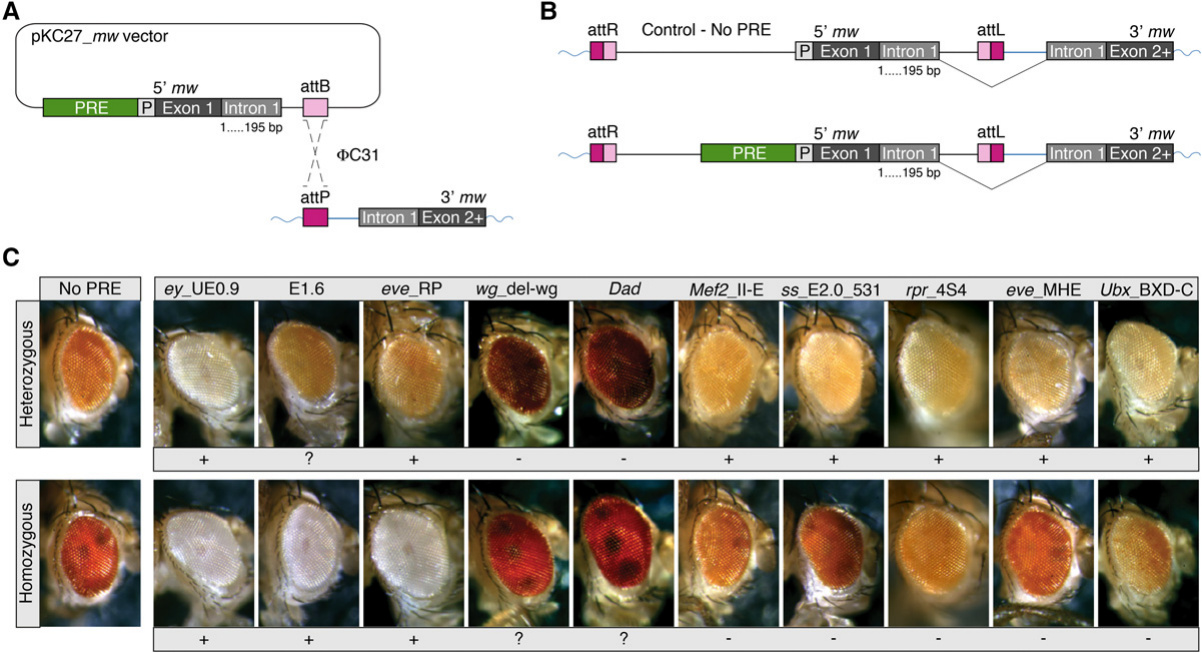
A subset of developmental enhancers mediates PSS. (*A*) An established split *mw* transgenic assay was used for PSS (Okulski et al. 2011): A donor vector (pKC27) containing a test PRE (in our case, an enhancer), the first exon of *mw*, and part of intron 1 (gray boxes; [P] *mw* promoter) was recombined into a genomic landing site (using ϕC31 integrase) that contains the rest of intron 1 and remaining exons of *mw*. (*B*) Upon successful site-specific integration, the *mw* gene was reconstituted, and orange/red eye color was restored. Integration at the same genomic site allowed eye color to be directly compared between age-matched flies with the control vector (*mw* reporter without PRE) and those with a PRE (green box). (*C*) The level of *mw* eye expression is indicated by eye color. All pictures were taken with the same settings on the same day for sibling pairs of 4-d-old adults. Comparing heterozygous eye color (across the *top* panels): Seven of the 10 enhancers have lighter eye color than the control (indicated by +). Comparing homozygous transgenic lines with their heterozygous siblings (cf. *top* and *bottom* panels for a given enhancer): (*C*) Three enhancers have a lighter eye color when homozygous compared with heterozygous, suggesting PSS; i.e., repression of the *mw* reporter gene (indicated by +). Five enhancers have darker eye color when homozygous, as for standard enhancers (indicated by −). In two enhancers, PSS is unclear (indicated by ?), as the heterozygous eye color is more brown than dark red. Enhancers tested were *ey*_UE0.9 (Adachi et al. 2003), E1.6 (Emmons et al. 2007), *eve*_RP (McDonald et al. 2003), *wg*_del-wg (Von Ohlen and Hooper 1997), *Dad* (Weiss et al. 2010), *Mef2*_II-E (Nguyen and Xu 1998), *ss*_E2.0_531 (Emmons et al. 2007), *rpr*_4S4 (Lohmann 2003), *eve*_MHE (Halfon et al. 2000; Knirr and Frasch 2001; Han et al. 2002), and *Ubx*_BXD-C (Christen and Bienz 1992).

First, we determined whether PhoRC-bound enhancers can function as PREs in vivo using two functional assays: (1) pairing-sensitive silencing (PSS) (Kassis 1994; Kassis and Brown 2013), which is a feature of most, although not all, characterized PREs, and (2) the ability to repress transcription in a PcG-dependent manner, an essential operational definition of a PRE (Americo et al. 2002; Kassis and Brown 2013). We used an established PSS assay based on the *mini-white* (*mw*) reporter, which does not contain any inherent enhancers and is therefore ideal to assay enhancers for PRE activity. Ten developmental enhancers with characterized spatio–temporal activity and occupancy of PhoRC were placed in front of a *mw* promoter in a construct where, upon successful integration of the plasmid into a specific landing site in the *Drosophila* genome, it reconstituted a functional *mw* reporter gene (Fig. 3A,B; Okulski et al. 2011). mw expression is clearly visible in the control transgenic line as a transition from the typical orange eye color in heterozygous flies carrying one copy of the *mw* gene to red eyes in homozygous flies carrying two copies (Fig. 3C). When a PRE is placed in front of the promoter, it can suppress transcriptional activity in the heterozygous state, and this silencing is even stronger in the homozygous state. Rather than going from orange to red eyes, homozygous flies with PSS therefore have a lighter eye color than their heterozygous siblings (Kassis 2002). While many of the previously characterized PREs mediate PSS, not all contain this function (Kassis 1994; Kassis 2002).

Seven out of 10 enhancers tested have lighter eye color when heterozygous compared with the heterozygous control without the putative PRE (Fig. 3C, top panels), the exceptions being the *wg*_del-wg and *Dad* enhancers. Three enhancers have even lighter eye color when homozygous compared with their heterozygous siblings, indicative of reporter gene silencing through PSS (*ey*_UE0.9 [Adachi et al. 2003], E1.6 [Emmons et al. 2007], and *eve*_RP [McDonald et al. 2003]) (Fig. 3C), while two additional enhancers (*wg*_del-wg [Von Ohlen and Hooper 1997] and *Dad* [Weiss et al. 2010]) may have PSS, although the results are less clear. Together, this indicates that a subset of enhancers likely contains the required regulatory information to act as PREs, as suggested previously for *eve*_RP (McDonald et al. 2003; Fujioka et al. 2008).

**Figure 4.**
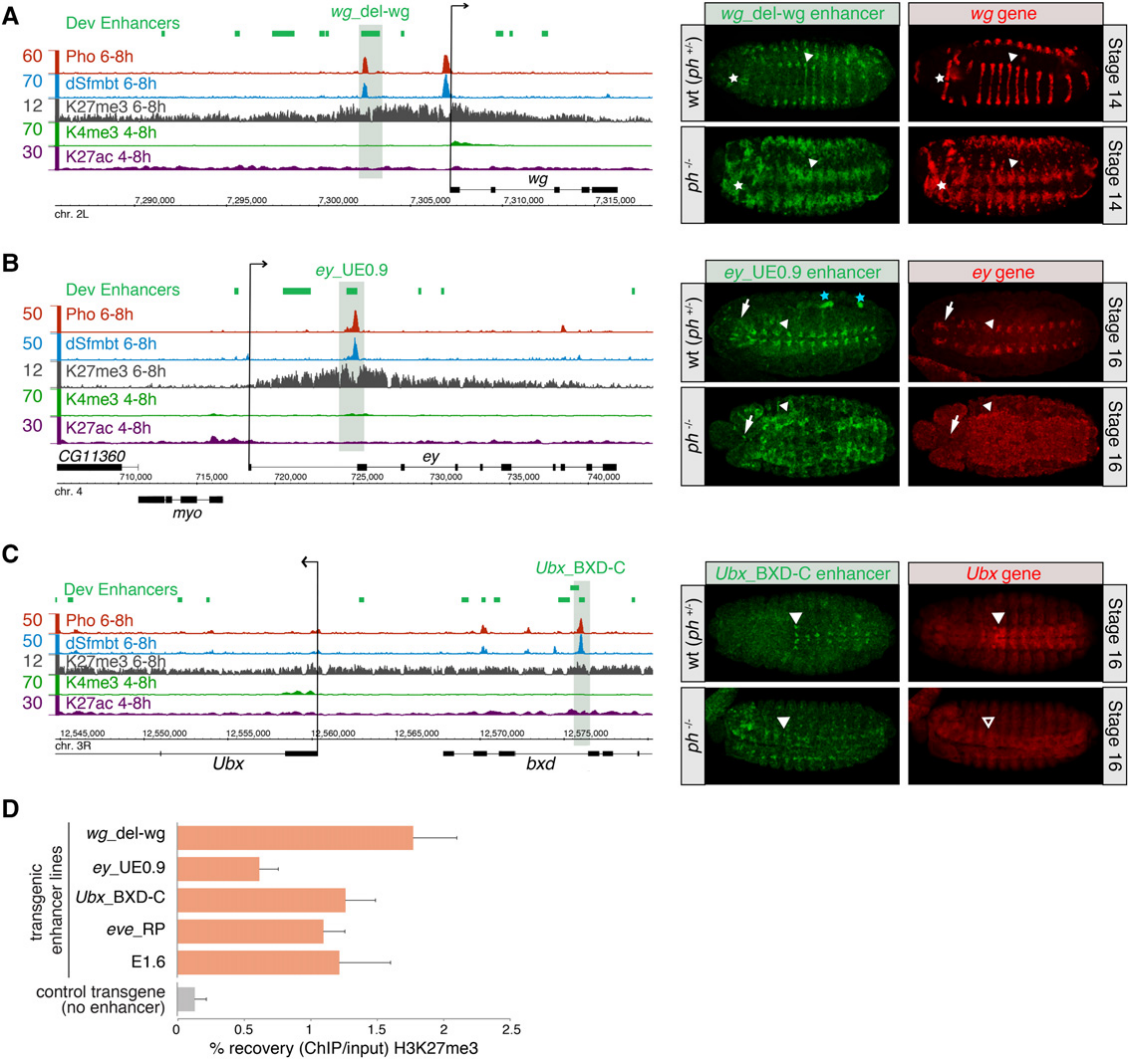
Developmental enhancers can function as PREs to mediate PcG-dependent silencing. (*A*–*C*) Genomic locus and activity of PhoRC-bound enhancers: *wg*_del-wg (*A*) (Von Ohlen and Hooper 1997), *ey*_UE0.9 (*B*) (Adachi et al. 2003), and *Ubx*_BXD-C (*C*) (Christen and Bienz 1992; Emmons et al. 2007). (*Left* panels) ChIP-seq signal for Pho (red), dSfmbt (blue; background subtracted), and H3K27me3 (Bonn et al. 2012a) from mesodermal cells and H3K4me3 and H3K27ac from whole embryos (modENCODE; H3 subtracted). Green boxes indicate developmental enhancers, and promoter arrows indicate the direction of the genes’ transcription. (*Right* panels) In situ hybridization against *mw* driven by the developmental enhancer (green) and the associated endogenous gene (red). Expression is shown in heterozygous *ph*^+/−^ (wild-type [wt]) and homozygous *ph*^−/−^ genetic backgrounds. In *ph*^−/−^ mutant embryos, enhancer activity is expanded for the *wg*_del-wg enhancer in ectodermal stripes (arrowhead) and the mandibular segment (asterisk; *A*), the *ey*_UE0.9 enhancer in the ventral nerve cord (arrowhead) and brain (arrow) (*B*), and the *Ubx*_BXD-C enhancer in the ventral nerve cord (*C*). The arrowhead marks the anterior expression boundary in the *ph*^+/−^ (wild-type) background, indicating the extent of anterior enhancer derepression in the *ph*^−/−^ mutant. The blue asterisk depicts background staining of the endogenous *white* gene (Fjose et al. 1984). Embryos are ventro–laterally (*A*) or ventrally (*B*,*C*) oriented with anterior to the left. (*D*) H3K27me3 ChIP-qPCR on chromatin isolated from embryos from five transgenic enhancer lines (orange) and a control transgenic line with empty vector (no enhancer; gray). The histogram shows the percentage recovery of ChIP over input; an average of two biological replicates was used. Error bars indicate standard errors of the mean.

To definitively show that these PhoRC-bound enhancers can function as PREs in vivo in a PcG-dependent manner, we placed the same 10 developmental enhancers tested above into a PRC1 loss-of-function mutant background by crossing the homozygous enhancer transgenes to a characterized *ph*-null allele (Parks et al. 2004; Feng et al. 2011). Enhancer activity was then examined in *ph* heterozygous and homozygous mutant backgrounds by in situ hybridization against the *mw* reporter in embryos at late stages of embryogenesis (when *ph* zygotic phenotypes are visible). Upon the removal of *ph*, the enhancer's activity was dramatically expanded in five out of 10 cases (50%), and therefore the maintenance of tissue-specific repression was lost (Fig. 4; Supplemental Fig. S5). Additionally, the corresponding endogenous gene activity was also disturbed as observed previously in a *ph* mutant background (Dura and Ingham 1988; Oktaba et al. 2008; Gambetta and Muller 2014). These elements therefore seem to act as an enhancer and a PRE for the same target gene.

For example, the *wg*-del-wg enhancer (Von Ohlen and Hooper 1997) upstream of the *wg* gene activates reporter gene expression in segmental ectodermal strips mirroring that of the gene's expression (Fig. 4A, top panels). In a *ph* mutant background, there is a dramatic expansion of the enhancer's activity along the anterio–posterior axis and head region, which recapitulates the effect of PcG removal on the endogenous *wg* gene (Fig. 4A). This element therefore acts as a developmental enhancer to activate expression in cells located in segmental stripes while acting as a PcG-dependent silencer of gene expression (i.e., a PRE) in the surrounding cells along the dorso–ventral axis.

Similarly, an enhancer in the first intron of the *ey* gene, *ey*_UE0.9 (Adachi et al. 2003), activates expression in a subset of neurons in the ventral nerve cord. In *ph* mutants, this enhancer's activity becomes dramatically derepressed, becoming active throughout what looks like the entire nervous system and parts of the peripheral nervous system (Fig. 4B). Although Pho is ubiquitously expressed, given the expression of endogenous *ph* in the nervous system (Deatrick 1992; de Camillis and Brock 1994; Fauvarque et al. 1995), this tissue may be more primed for derepression in *ph* mutants. A similar PcG-mediated silencing was observed for the *eve*_RP (McDonald et al. 2003) and E1.6 (Emmons et al. 2007) enhancers, both of which are strongly derepressed in the nervous system in *ph*^−/−^ mutants (Supplemental Fig. S5a,b). The well-characterized *Ubx*_BXD-C enhancer (Christen and Bienz 1992), located ∼15 kb upstream of the *Ubx* gene within the intron of *bxd*, drives expression in a subset of neuroblasts in the ventral nerve cord in abdominal segments matching that of *Ubx* expression (Akam and Martinez-Arias 1985). In *ph* mutants, both the enhancer and the gene become derepressed within more anterior regions of the mesoderm and ectoderm, cells where the enhancer and *Ubx* gene are normally never active (Fig. 4C).

For these five enhancer elements with clear PRE activity (*wg-*del-wg, *ey*_UE0.9, *Ubx* BxD-C, *eve*-RP, and E1.6), we next determined whether they are sufficient to recruit the Polycomb system to the transgenic enhancer de novo. Embryos were collected from each of the five transgenic reporter lines in addition to a transgenic line with an integrated empty vector and used for ChIP-qPCR against H3K27me3 as an indicator of PRC2 recruitment. H3K27me3 was detected using primers that specifically amplify signal from the integrated transgenic enhancer. The ChIPs on all five transgenic enhancer lines indicate that H3K27me3 is highly enriched on the integrated enhancer transgenes, which is not the case in the transgenic line with the empty vector (Fig. 4D). These enhancers are therefore sufficient to recruit PRC2 activity de novo, supporting our functional data that they can act as dual-function enhancer/PREs.

Taken together, these three inherent properties (direct recruitment of the PcG system to the enhancer, PSS, and genetic dependence on PcG for silencing) indicate that a subset of characterized developmental enhancers can function as PREs in a PcG-dependent manner. The latter two requirements are fulfilled in five out of 10 (50%) enhancers tested, suggesting that PcG-bound enhancers may be PcG-responsive (i.e. able to silence *mw* reporter gene expression) only in specific permissive contexts. This may reflect the inherent properties of each enhancer's sequence, its potential to interact with the local genomic environment such as nearby silencers, or the relative levels of PhoRC occupancy. The latter, for example, may explain our results in the *eve* locus, where we tested two enhancers for PRE activity: the neuronal *eve*_RP enhancer (Supplemental Fig. S5a), which has high levels of PhoRC occupancy and is PcG-responsive (Supplemental Fig. S5a), and the muscle–heart *eve*_MHE enhancer, which has a smaller PhoRC peak and cannot function as a PRE, at least in this transgenic context (Supplemental Fig. S5c). In fact, rather than being silenced by polycomb, the MHE enhancer appears to lose activity in the visceral muscle in *ph* mutants (Supplemental Fig. S5c), suggesting that it may even be positively influenced by PcG.

### Operational PREs can function as developmental enhancers in vivo

**Figure 5.**
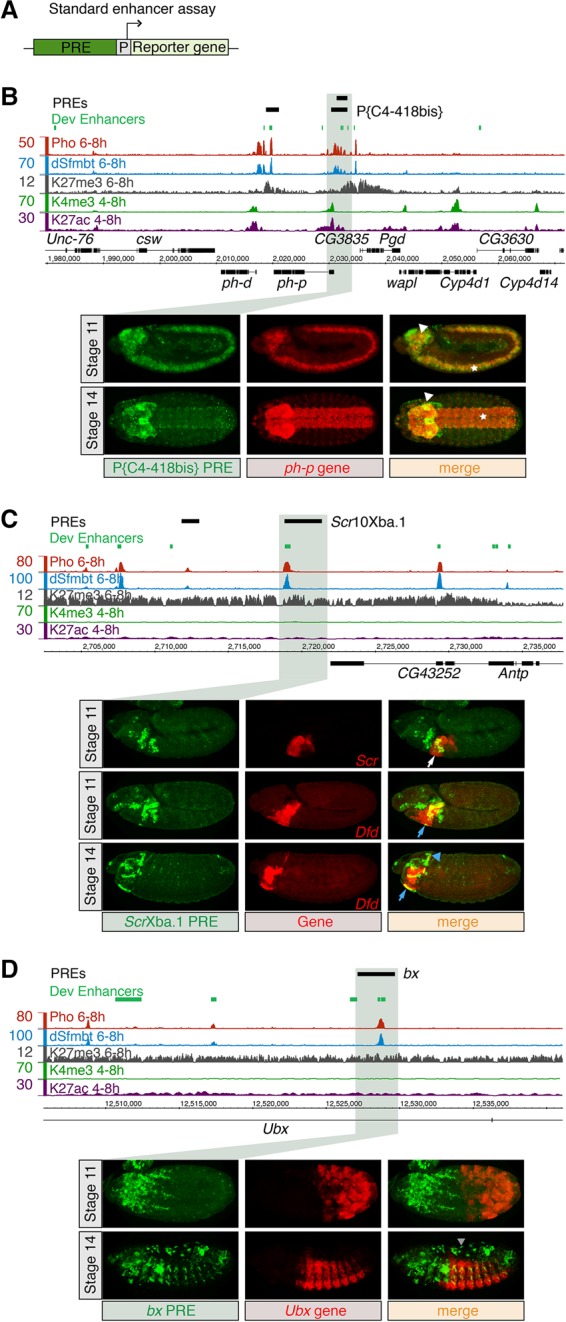
PREs can function as developmental enhancers to activate spatio–temporal expression. (*A*) Standard enhancer transgenic assay in which an enhancer (in our case, a PRE; dark green) is placed in front of a minimal promoter (gray box; [P] promoter) and *lacZ* reporter gene (light-green box) and stably integrated into the same genomic location using ϕC31 integrase. (*B*–*D*) Genomic locus and activity of characterized PREs: (*Top* panels) ChIP-seq signal for Pho (red) and dSfmbt (blue; background subtracted), H3K27me3 (Bonn et al. 2012a) from mesodermal cells, and H3K4me3 and H3K27ac from whole embryos (modENCODE; H3 subtracted). Black boxes indicate characterized PREs: P{C4-418bis} PRE (*B*) (Bloyer et al. 2003), *Scr*10Xba.1 PRE (*C*) (Gindhart and Kaufman 1995; Kapoun and Kaufman 1995; Ringrose et al. 2003), and *bx* PRE (*D*) (Orlando et al. 1998). (*Bottom* panels) Double in situ hybridization against the *lacZ* reporter gene driven by the characterized PRE (green) and the associated endogenous gene (red). Expression driven by P{C4-418bis}, *Scr*10Xba.1, and *bx* PREs extensively (*B*) or partially (*C*,*D*) overlaps that of the endogenous gene in the developing brain (white arrowhead) and ventral nerve cord (asterisk) (*B*); maxillary (blue arrow), labial (white arrow), and optical lobes (blue arrowhead) (*C*); and amnioserosa (gray arrowhead) (*D*). Embryos are laterally (*B* [stage 11], *C*,*D*) or dorsally (*B*; stage 14) oriented with anterior to the left.

Having demonstrated that a subset of developmental enhancers can also function as PREs, we next assessed whether classic operationally defined *Drosophila* PREs can function as developmental enhancers in vivo. Our hypothesis is that these PREs would act as developmental enhancers in another tissue. Rather than a standard PRE reporter construct (which contains an enhancer linked to a promoter) (Americo et al. 2002), we used a standard transgenic reporter construct used to test enhancer activity, which contains a minimal promoter and a *lacZ* reporter (Fig. 5A). Six well-characterized PREs were placed in front of the *hsp70* minimal promoter and stably integrated into the same genomic location using the ϕC31 integrase system (Fig. 5A; Bischof et al. 2007). Enhancer activity from the PRE was assayed using in situ hybridization against the *lacZ* reporter. By comparing enhancer activity with the spatio–temporal pattern of the PRE-associated genes, we also assessed whether PREs can enhance the gene that they repress. Four of the six tested elements were sufficient to function as an enhancer in vivo, activating tissue-specific expression, with three having overlapping activity with the PRE's associated target gene during embryonic development (Fig. 5; Supplemental Fig. S6). For example, the PcG-dependent PRE P{C4-418bis} (Bloyer et al. 2003) is sufficient to activate strong expression throughout the developing ventral nerve cord and brain at stages 11 and 12 (Fig. 5B), where it recapitulates the expression of the endogenous PcG gene *ph-p* (Deatrick 1992; de Camillis and Brock 1994; Fauvarque et al. 1995). Similarly, a PRE within the *Antennapedia Hox* complex, *Scr*10Xba.1 PRE, acts to silence the *Scr* gene's expression outside of its normal expression domain in a PcG-dependent manner (Gindhart and Kaufman 1995; Kapoun and Kaufman 1995; Ringrose et al. 2003). Here we show that this element is sufficient to activate transcription, recapitulating part of the spatio–temporal expression of the endogenous *Scr* gene in the labial lobe and the nearby *Dfd* gene in the maxillary and optical lobes (Fig. 5C). The *bx* PRE in the *bithorax* complex (Orlando et al. 1998), the second major *Hox* cluster, activates expression in the amnioserosa, a pattern also observed at much lower levels for the endogenous gene *Ubx* (Fig. 5D; Akam and Martinez-Arias 1985). The bulk of activated expression is in more anterior regions that do not overlap the expression of *Ubx*, suggesting that this element acts as a PRE for *Ubx* and an enhancer for a different gene.

Taken together, these results indicate that, in different developmental contexts, the same regulatory element can confer either an enhancer or a PRE activity, suggesting dual potential of a subset of regulatory elements depending on the context of recruited factors. Hence, fine-tuning the interplay between tissue-specific TFs and PcG proteins on these PhoRC enhancers likely switches their function from activating spatio–temporal activity (enhancer) to stably maintaining a silenced state (PRE).

## Discussion

While enhancers initiate spatio–temporal transcriptional activity, PREs maintain a previously determined transcriptional state of their target genes, thus leading to transcriptional memory (Schuettengruber et al. 2011; Bauer et al. 2016). PREs are generally thought to be dedicated solely to gene silencing and not to contain enhancer-like features to activate gene expression (Kassis and Muller 2015). Here we present evidence to the contrary, that both functions can be encoded in the same *cis-*regulatory element, depending on the cellular context. This is not a rare event—almost 25% of PhoRC occupancy is at developmental enhancers. Of the 16 elements that we tested experimentally (either enhancers for PRE activity or PREs for enhancer activity), nine have dual function, being sufficient to activate transcription in a specific spatio–temporal pattern and mediate PcG-dependent silencing in vivo.

**Figure 6.**
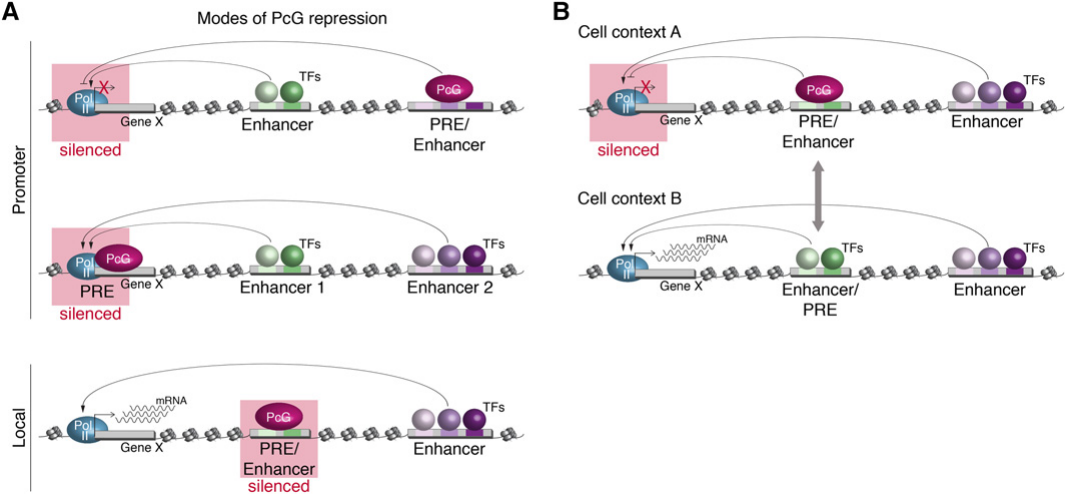
Schematic model for the action of dual elements. (*A*) PREs can silence gene expression by acting at a distance within a three-dimensional topology that silences promoter activity (*top* panel) or directly at the promoter (*middle* panel) to silence transcription. (*Bottom* panel) Our data suggest a third mode, where PcG acts locally to silence a PRE/enhancer dual-element activity, leaving the promoter still available for activation by another enhancer (see also Fig. 1F). (*B*) A dual element can act as a PRE (*top* panel) in one cell context through the recruitment of the PcG system (purple ellipsoid) to silence gene expression, while, in another context, the same element can act as an enhancer (*bottom* panel) through the recruitment of tissue-specific TFs (green spheres) to initiate spatio–temporal gene expression.

These dual elements have interesting implications for transcriptional regulation during embryonic development. First, at the level of PcG protein recruitment, this subset of enhancers is highly enriched in the Pho motif, which distinguishes them from other developmental enhancers. This suggests that the recruitment of Pho to PhoRC enhancers is direct via sequence-specific DNA binding, consistent with an instructive model of recruitment (Klose et al. 2013), although other factors are likely involved. PcG proteins and developmental TFs bind in close proximity to each other within the same element (a single DNase hypersensitive site), raising the possibility of direct interplay between the two. Our results indicate that the activity of PhoRC-bound enhancers is dominated by tissue-specific TFs that activate transcription in some cells while being dominated by a functional PcG complex in other cells (Fig. 6). Is this due to mutually exclusive occupancy of developmental TFs and PcG proteins in different tissues, or do they compete functionally at these elements? The dramatic derepression of enhancer activity in different cell types upon PcG protein removal suggests that other tissue-specific TFs must occupy these enhancers in the PcG silenced cell. This has interesting implications for enhancer activity, as it is well known that TFs bind to thousands of sites (tens of thousands in mammalian cells), but only a subset of associated target genes changes expression when the TF is removed. This has led to the general assumption that the majority of binding events is nonfunctional or neutral. Our data suggest that at least a subset of this embryonic occupancy can be functional if not actively antagonized by the presence of PcGs.

Second, enhancer-mediated polycomb recruitment has interesting implications for the mechanism of PcG-mediated silencing. The current models suggest that PcG proteins silence transcription mainly by silencing a gene's promoter (Papp and Muller 2006), in keeping with PcG recruitment to CpG islands in vertebrates, or by coordinating a three-dimensional repressive topology, where the entire gene's locus is silenced (Bantignies and Cavalli 2011; Simon and Kingston 2013). In either mode, a gene's promoter would not be permissive to enhancer activation. Our data suggest that there may be a third mode of very local silencing at an individual enhancer, leaving the promoter and the rest of the gene's regulatory landscape open for activation by other enhancers, as we observed at the *prat2* locus (Fig. 1F). This would allow for much more fine-tuning of silencing in individual tissues and stages. It also suggests that PcG proteins could play a more dynamic role, similar to a “standard” transcriptional repressor at enhancers.

Third, this may have broader implications for cell fate decisions during rapid developmental transitions. When multipotent cells become specified into different lineages, a specific transcriptional program often needs to be activated in one cell while being repressed in other cells from the same progenitor population (Ciglar et al. 2014). Having active enhancers in the precursor cells remain accessible to directly recruit the PcG complexes would ensure that these enhancers become silenced in a timely manner. Conversely, having maternally deposited PcG proteins already bound to enhancers early in development may serve as placeholders to ensure that these dual elements remain open and available for TFs to activate at the appropriate development stage. Interestingly, in the majority of the tested cases, PcG proteins and developmental TFs use these dual elements to regulate the same target gene (Fig. 4; Supplemental Fig. S5), the vast majority of which is key developmental regulators of cell identity.

The identification of PREs in other species has remained a key challenge, with only a handful of PREs identified in mammals (Bauer et al. 2016) and plants (Xiao and Wagner 2015) to date. In mammals, the PcG system is recruited to inactive CpG islands (Di Croce and Helin 2013; Riising et al. 2014), with few specific sequence features. Although there are mammalian homologs of the *Drosophila* Pho and dSfmbt proteins, Yin Yang 1 (YY1) and SFMBT, respectively, the conservation of PhoRC as a complex and its involvement in mammalian PcG silencing remain unclear (Bauer et al. 2016). We propose that such dual enhancers/PREs will also exist in mammals, although, given this apparent lack of conservation of YY1 function, their mechanism of PcG recruitment may have diverged.

## Materials and methods

Genome-wide binding profiles for Pho and dSfmbt in mesodermal embryonic nuclei were obtained using BiTS-ChIP (Bonn et al. 2012b) using characterized antibodies for both proteins (Klymenko et al. 2006). ChIP was performed at two consecutive time points for Pho (4–6 h and 6–8 h after egg laying [AEL]) and one for dSfmbt (6–8 h AEL), with two independent biological replicates for each condition. Libraries were amplified from 2–3 ng of immunoprecipitated material and sequenced on either an Illumina GA_IIx (Pho) or Hi-Seq (dSfmbt) machine. The data were analyzed as described in the Supplemental Material. Transgenic assays were carried out in vivo on characterized enhancers using a PSS assay in *Drosophila* eyes and in the mutant genetic background in embryos using in situ hybridization. Operational PREs were tested for enhancer activity in vivo in transgenic reporter assays using in situ hybridization. All raw data have been submitted to the EBI ArrayExpress and European Nucleotide Archive (ENA) databases under accession numbers E-MTAB-4585 and ERP000560, and processed data are provided on the Furlong laboratory's Web site (http://furlonglab.embl.de/data).

## Supplementary Material

Supplemental Material

## References

[ERCEGGAD292870C1] Adachi Y, Hauck B, Clements J, Kawauchi H, Kurusu M, Totani Y, Kang YY, Eggert T, Walldorf U, Furukubo-Tokunaga K, 2003 Conserved *cis*-regulatory modules mediate complex neural expression patterns of the eyeless gene in the *Drosophila* brain. Mech Dev 120: 1113–1126. 14568101 10.1016/j.mod.2003.08.007

[ERCEGGAD292870C2] Akam ME, Martinez-Arias A. 1985 The distribution of ultrabithorax transcripts in *Drosophila* embryos. EMBO J 4: 1689–1700. 16453626 10.1002/j.1460-2075.1985.tb03838.x PMC554405

[ERCEGGAD292870C3] Americo J, Whiteley M, Brown JL, Fujioka M, Jaynes JB, Kassis JA. 2002 A complex array of DNA-binding proteins required for pairing-sensitive silencing by a polycomb group response element from the *Drosophila* engrailed gene. Genetics 160: 1561–1571. 11973310 10.1093/genetics/160.4.1561 PMC1462036

[ERCEGGAD292870C4] Bantignies F, Cavalli G. 2011 Polycomb group proteins: repression in 3D. Trends Genet 27: 454–464. 21794944 10.1016/j.tig.2011.06.008

[ERCEGGAD292870C5] Bauer M, Trupke J, Ringrose L. 2016 The quest for mammalian Polycomb response elements: are we there yet? Chromosoma 125: 471–496. 26453572 10.1007/s00412-015-0539-4 PMC4901126

[ERCEGGAD292870C6] Beisel C, Paro R. 2011 Silencing chromatin: comparing modes and mechanisms. Nat Rev Genet 12: 123–135. 21221116 10.1038/nrg2932

[ERCEGGAD292870C7] Bischof J, Maeda RK, Hediger M, Karch F, Basler K. 2007 An optimized transgenesis system for *Drosophila* using germ-line-specific phiC31 integrases. Proc Natl Acad Sci 104: 3312–3317. 17360644 10.1073/pnas.0611511104 PMC1805588

[ERCEGGAD292870C8] Bloyer S, Cavalli G, Brock HW, Dura JM. 2003 Identification and characterization of polyhomeotic PREs and TREs. Dev Biol 261: 426–442. 14499651 10.1016/s0012-1606(03)00314-2

[ERCEGGAD292870C9] Bonn S, Zinzen RP, Girardot C, Gustafson EH, Perez-Gonzalez A, Delhomme N, Ghavi-Helm Y, Wilczynski B, Riddell A, Furlong EE. 2012a Tissue-specific analysis of chromatin state identifies temporal signatures of enhancer activity during embryonic development. Nat Genet 44: 148–156. 22231485 10.1038/ng.1064

[ERCEGGAD292870C10] Bonn S, Zinzen RP, Perez-Gonzalez A, Riddell A, Gavin AC, Furlong EE. 2012b Cell type-specific chromatin immunoprecipitation from multicellular complex samples using BiTS-ChIP. Nat Protoc 7: 978–994. 22538849 10.1038/nprot.2012.049

[ERCEGGAD292870C11] Brown JL, Mucci D, Whiteley M, Dirksen ML, Kassis JA. 1998 The *Drosophila* Polycomb group gene pleiohomeotic encodes a DNA binding protein with homology to the transcription factor YY1. Mol Cell 1: 1057–1064. 9651589 10.1016/s1097-2765(00)80106-9

[ERCEGGAD292870C12] Cannavo E, Khoueiry P, Garfield DA, Geeleher P, Zichner T, Gustafson EH, Ciglar L, Korbel JO, Furlong EE. 2015 Shadow enhancers are pervasive features of developmental regulatory networks. Curr Biol 26: 38–51. 26687625 10.1016/j.cub.2015.11.034 PMC4712172

[ERCEGGAD292870C13] Christen B, Bienz M. 1992 A *cis*-element mediating ultrabithorax autoregulation in the central nervous system. Mech Dev 39: 73–80. 1362651 10.1016/0925-4773(92)90027-h

[ERCEGGAD292870C14] Ciglar L, Girardot C, Wilczynski B, Braun M, Furlong EE. 2014 Coordinated repression and activation of two transcriptional programs stabilizes cell fate during myogenesis. Development 141: 2633–2643. 24961800 10.1242/dev.101956 PMC4146391

[ERCEGGAD292870C15] Creyghton MP, Cheng AW, Welstead GG, Kooistra T, Carey BW, Steine EJ, Hanna J, Lodato MA, Frampton GM, Sharp PA, 2010 Histone H3K27ac separates active from poised enhancers and predicts developmental state. Proc Natl Acad Sci 107: 21931–21936. 21106759 10.1073/pnas.1016071107 PMC3003124

[ERCEGGAD292870C16] Czermin B, Melfi R, McCabe D, Seitz V, Imhof A, Pirrotta V. 2002 *Drosophila* enhancer of Zeste/ESC complexes have a histone H3 methyltransferase activity that marks chromosomal Polycomb sites. Cell 111: 185–196. 12408863 10.1016/s0092-8674(02)00975-3

[ERCEGGAD292870C17] Deatrick J. 1992 Localization in situ of polyhomeotic transcripts in *Drosophila* embryos reveals spatially restricted expression beginning at the blastoderm stage. Dev Genet 13: 326–330. 1363400 10.1002/dvg.1020130503

[ERCEGGAD292870C18] de Camillis MA, Brock HW. 1994 Expression of the Polyhomeotic locus in development of *Drosophila melanogaster*. Rouxs Arch Dev Biol 203: 429–438. 28305949 10.1007/BF00188692

[ERCEGGAD292870C19] Dejardin J, Rappailles A, Cuvier O, Grimaud C, Decoville M, Locker D, Cavalli G. 2005 Recruitment of *Drosophila* Polycomb group proteins to chromatin by DSP1. Nature 434: 533–538. 15791260 10.1038/nature03386

[ERCEGGAD292870C20] DeVido SK, Kwon D, Brown JL, Kassis JA. 2008 The role of Polycomb-group response elements in regulation of engrailed transcription in *Drosophila*. Development 135: 669–676. 18199580 10.1242/dev.014779

[ERCEGGAD292870C21] Di Croce L, Helin K. 2013 Transcriptional regulation by Polycomb group proteins. Nat Struct Mol Biol 20: 1147–1155. 24096405 10.1038/nsmb.2669

[ERCEGGAD292870C22] Duncan IM. 1982 Polycomblike: a gene that appears to be required for the normal expression of the bithorax and antennapedia gene complexes of *Drosophila melanogaster*. Genetics 102: 49–70. 6813190 10.1093/genetics/102.1.49 PMC1201924

[ERCEGGAD292870C23] Dura JM, Ingham P. 1988 Tissue- and stage-specific control of homeotic and segmentation gene expression in *Drosophila* embryos by the polyhomeotic gene. Development 103: 733–741. 2907878 10.1242/dev.103.4.733

[ERCEGGAD292870C24] Emmons RB, Duncan D, Duncan I. 2007 Regulation of the *Drosophila* distal antennal determinant spineless. Dev Biol 302: 412–426. 17084833 10.1016/j.ydbio.2006.09.044 PMC1876787

[ERCEGGAD292870C25] Enderle D, Beisel C, Stadler MB, Gerstung M, Athri P, Paro R. 2011 Polycomb preferentially targets stalled promoters of coding and noncoding transcripts. Genome Res 21: 216–226. 21177970 10.1101/gr.114348.110 PMC3032925

[ERCEGGAD292870C26] Fauvarque MO, Zuber V, Dura JM. 1995 Regulation of polyhomeotic transcription may involve local changes in chromatin activity in *Drosophila*. Mech Dev 52: 343–355. 8541220 10.1016/0925-4773(95)00412-t

[ERCEGGAD292870C27] Feng S, Huang J, Wang J. 2011 Loss of the Polycomb group gene polyhomeotic induces non-autonomous cell overproliferation. EMBO Rep 12: 157–163. 21164514 10.1038/embor.2010.188 PMC3049426

[ERCEGGAD292870C28] Fjose A, Polito LC, Weber U, Gehring WJ. 1984 Developmental expression of the white locus of *Drosophila melanogaster*. EMBO J 3: 2087–2094. 16453550 10.1002/j.1460-2075.1984.tb02095.x PMC557647

[ERCEGGAD292870C29] Frey F, Sheahan T, Finkl K, Stoehr G, Mann M, Benda C, Muller J. 2016 Molecular basis of PRC1 targeting to Polycomb response elements by PhoRC. Genes Dev 30: 1116–1127. 27151979 10.1101/gad.279141.116 PMC4863741

[ERCEGGAD292870C30] Fritsch C, Brown JL, Kassis JA, Muller J. 1999 The DNA-binding polycomb group protein pleiohomeotic mediates silencing of a *Drosophila* homeotic gene. Development 126: 3905–3913. 10433918 10.1242/dev.126.17.3905

[ERCEGGAD292870C31] Fujioka M, Yusibova GL, Zhou J, Jaynes JB. 2008 The DNA-binding Polycomb-group protein pleiohomeotic maintains both active and repressed transcriptional states through a single site. Development 135: 4131–4139. 19029043 10.1242/dev.024554 PMC2710970

[ERCEGGAD292870C32] Gaertner B, Johnston J, Chen K, Wallaschek N, Paulson A, Garruss AS, Gaudenz K, De Kumar B, Krumlauf R, Zeitlinger J. 2012 Poised RNA polymerase II changes over developmental time and prepares genes for future expression. Cell Rep 2: 1670–1683. 23260668 10.1016/j.celrep.2012.11.024 PMC3572839

[ERCEGGAD292870C33] Gallo SM, Gerrard DT, Miner D, Simich M, Des Soye B, Bergman CM, Halfon MS. 2011 REDfly v3.0: toward a comprehensive database of transcriptional regulatory elements in *Drosophila*. Nucleic Acids Res 39: D118–D123. 20965965 10.1093/nar/gkq999 PMC3013816

[ERCEGGAD292870C34] Gambetta MC, Muller J. 2014 O-GlcNAcylation prevents aggregation of the Polycomb group repressor polyhomeotic. Dev Cell 31: 629–639. 25468754 10.1016/j.devcel.2014.10.020

[ERCEGGAD292870C35] Geisler SJ, Paro R. 2015 Trithorax and Polycomb group-dependent regulation: a tale of opposing activities. Development 142: 2876–2887. 26329598 10.1242/dev.120030

[ERCEGGAD292870C36] Gindhart JG Jr, Kaufman TC. 1995 Identification of Polycomb and trithorax group responsive elements in the regulatory region of the *Drosophila* homeotic gene Sex combs reduced. Genetics 139: 797–814. 7713433 10.1093/genetics/139.2.797 PMC1206382

[ERCEGGAD292870C37] Halfon MS, Carmena A, Gisselbrecht S, Sackerson CM, Jimenez F, Baylies MK, Michelson AM. 2000 Ras pathway specificity is determined by the integration of multiple signal-activated and tissue-restricted transcription factors. Cell 103: 63–74. 11051548 10.1016/s0092-8674(00)00105-7

[ERCEGGAD292870C38] Han Z, Fujioka M, Su M, Liu M, Jaynes JB, Bodmer R. 2002 Transcriptional integration of competence modulated by mutual repression generates cell-type specificity within the cardiogenic mesoderm. Dev Biol 252: 225–240. 12482712 10.1006/dbio.2002.0846 PMC2693947

[ERCEGGAD292870C39] Junion G, Spivakov M, Girardot C, Braun M, Gustafson EH, Birney E, Furlong EE. 2012 A transcription factor collective defines cardiac cell fate and reflects lineage history. Cell 148: 473–486. 22304916 10.1016/j.cell.2012.01.030

[ERCEGGAD292870C40] Kapoun AM, Kaufman TC. 1995 Regulatory regions of the homeotic gene proboscipedia are sensitive to chromosomal pairing. Genetics 140: 643–658. 7498743 10.1093/genetics/140.2.643 PMC1206641

[ERCEGGAD292870C41] Kassis JA. 1994 Unusual properties of regulatory DNA from the *Drosophila* engrailed gene: three ‘pairing-sensitive’ sites within a 1.6-kb region. Genetics 136: 1025–1038. 8005412 10.1093/genetics/136.3.1025 PMC1205860

[ERCEGGAD292870C42] Kassis JA. 2002 Pairing-sensitive silencing, polycomb group response elements, and transposon homing in *Drosophila*. Adv Genet 46: 421–438. 11931233 10.1016/s0065-2660(02)46015-4

[ERCEGGAD292870C43] Kassis JA, Brown JL. 2013 Polycomb group response elements in *Drosophila* and vertebrates. Adv Genet 81: 83–118. 23419717 10.1016/B978-0-12-407677-8.00003-8 PMC4157523

[ERCEGGAD292870C44] Kassis JA, Muller J. 2015 Transcription through Polycomb response elements does not induce a switch from repression to activation. Proc Natl Acad Sci 112: 14755–14756. 26567151 10.1073/pnas.1520102112 PMC4672789

[ERCEGGAD292870C45] Klose RJ, Cooper S, Farcas AM, Blackledge NP, Brockdorff N. 2013 Chromatin sampling—an emerging perspective on targeting polycomb repressor proteins. PLoS Genet 9: e1003717. 23990804 10.1371/journal.pgen.1003717 PMC3749931

[ERCEGGAD292870C46] Klymenko T, Muller J. 2004 The histone methyltransferases Trithorax and Ash1 prevent transcriptional silencing by Polycomb group proteins. EMBO Rep 5: 373–377. 15031712 10.1038/sj.embor.7400111 PMC1299022

[ERCEGGAD292870C47] Klymenko T, Papp B, Fischle W, Kocher T, Schelder M, Fritsch C, Wild B, Wilm M, Muller J. 2006 A Polycomb group protein complex with sequence-specific DNA-binding and selective methyl-lysine-binding activities. Genes Dev 20: 1110–1122. 16618800 10.1101/gad.377406 PMC1472471

[ERCEGGAD292870C48] Knirr S, Frasch M. 2001 Molecular integration of inductive and mesoderm-intrinsic inputs governs even-skipped enhancer activity in a subset of pericardial and dorsal muscle progenitors. Dev Biol 238: 13–26. 11783990 10.1006/dbio.2001.0397

[ERCEGGAD292870C49] Kvon EZ, Kazmar T, Stampfel G, Yanez-Cuna JO, Pagani M, Schernhuber K, Dickson BJ, Stark A. 2014 Genome-scale functional characterization of *Drosophila* developmental enhancers in vivo. Nature 512: 91–95. 24896182 10.1038/nature13395

[ERCEGGAD292870C50] Kwong C, Adryan B, Bell I, Meadows L, Russell S, Manak JR, White R. 2008 Stability and dynamics of polycomb target sites in *Drosophila* development. PLoS Genet 4: e1000178. 18773083 10.1371/journal.pgen.1000178 PMC2525605

[ERCEGGAD292870C51] Lewis EB. 1978 A gene complex controlling segmentation in *Drosophila*. Nature 276: 565–570. 103000 10.1038/276565a0

[ERCEGGAD292870C52] Lohmann I. 2003 Dissecting the regulation of the *Drosophila* cell death activator reaper. Gene Expr Patterns 3: 159–163. 12711543 10.1016/s1567-133x(03)00008-5

[ERCEGGAD292870C53] McDonald JA, Fujioka M, Odden JP, Jaynes JB, Doe CQ. 2003 Specification of motoneuron fate in *Drosophila*: integration of positive and negative transcription factor inputs by a minimal eve enhancer. J Neurobiol 57: 193–203. 14556285 10.1002/neu.10264

[ERCEGGAD292870C54] Muller J, Kassis JA. 2006 Polycomb response elements and targeting of Polycomb group proteins in *Drosophila*. Curr Opin Genet Dev 16: 476–484. 16914306 10.1016/j.gde.2006.08.005

[ERCEGGAD292870C55] Muller J, Verrijzer P. 2009 Biochemical mechanisms of gene regulation by polycomb group protein complexes. Curr Opin Genet Dev 19: 150–158. 19345089 10.1016/j.gde.2009.03.001

[ERCEGGAD292870C56] Muller J, Hart CM, Francis NJ, Vargas ML, Sengupta A, Wild B, Miller EL, O'Connor MB, Kingston RE, Simon JA. 2002 Histone methyltransferase activity of a *Drosophila* Polycomb group repressor complex. Cell 111: 197–208. 12408864 10.1016/s0092-8674(02)00976-5

[ERCEGGAD292870C57] Negre N, Hennetin J, Sun LV, Lavrov S, Bellis M, White KP, Cavalli G. 2006 Chromosomal distribution of PcG proteins during *Drosophila* development. PLoS Biol 4: e170. 16613483 10.1371/journal.pbio.0040170 PMC1440717

[ERCEGGAD292870C58] Nguyen HT, Xu X. 1998 *Drosophila* mef2 expression during mesoderm development is controlled by a complex array of cis-acting regulatory modules. Dev Biol 204: 550–566. 9882489 10.1006/dbio.1998.9081

[ERCEGGAD292870C59] Oktaba K, Gutierrez L, Gagneur J, Girardot C, Sengupta AK, Furlong EE, Muller J. 2008 Dynamic regulation by polycomb group protein complexes controls pattern formation and the cell cycle in *Drosophila*. Dev Cell 15: 877–889. 18993116 10.1016/j.devcel.2008.10.005

[ERCEGGAD292870C60] Okulski H, Druck B, Bhalerao S, Ringrose L. 2011 Quantitative analysis of polycomb response elements (PREs) at identical genomic locations distinguishes contributions of PRE sequence and genomic environment. Epigenetics Chromatin 4: 4. 21410956 10.1186/1756-8935-4-4 PMC3070613

[ERCEGGAD292870C61] Orlando V, Jane EP, Chinwalla V, Harte PJ, Paro R. 1998 Binding of trithorax and Polycomb proteins to the bithorax complex: dynamic changes during early *Drosophila* embryogenesis. EMBO J 17: 5141–5150. 9724650 10.1093/emboj/17.17.5141 PMC1170842

[ERCEGGAD292870C62] Papp B, Muller J. 2006 Histone trimethylation and the maintenance of transcriptional ON and OFF states by trxG and PcG proteins. Genes Dev 20: 2041–2054. 16882982 10.1101/gad.388706 PMC1536056

[ERCEGGAD292870C63] Parks AL, Cook KR, Belvin M, Dompe NA, Fawcett R, Huppert K, Tan LR, Winter CG, Bogart KP, Deal JE, 2004 Systematic generation of high-resolution deletion coverage of the *Drosophila melanogaster* genome. Nat Genet 36: 288–292. 14981519 10.1038/ng1312

[ERCEGGAD292870C64] Pengelly AR, Copur O, Jackle H, Herzig A, Muller J. 2013 A histone mutant reproduces the phenotype caused by loss of histone-modifying factor Polycomb. Science 339: 698–699. 23393264 10.1126/science.1231382

[ERCEGGAD292870C65] Piunti A, Shilatifard A. 2016 Epigenetic balance of gene expression by Polycomb and COMPASS families. Science 352: aad9780. 27257261 10.1126/science.aad9780

[ERCEGGAD292870C66] Rada-Iglesias A, Bajpai R, Swigut T, Brugmann SA, Flynn RA, Wysocka J. 2011 A unique chromatin signature uncovers early developmental enhancers in humans. Nature 470: 279–283. 21160473 10.1038/nature09692 PMC4445674

[ERCEGGAD292870C67] Ray P, De S, Mitra A, Bezstarosti K, Demmers JA, Pfeifer K, Kassis JA. 2016 Combgap contributes to recruitment of Polycomb group proteins in *Drosophila*. Proc Natl Acad Sci 113: 3826–3831. 27001825 10.1073/pnas.1520926113 PMC4833261

[ERCEGGAD292870C68] Riising EM, Comet I, Leblanc B, Wu X, Johansen JV, Helin K. 2014 Gene silencing triggers polycomb repressive complex 2 recruitment to CpG islands genome wide. Mol Cell 55: 347–360. 24999238 10.1016/j.molcel.2014.06.005

[ERCEGGAD292870C69] Ringrose L, Rehmsmeier M, Dura JM, Paro R. 2003 Genome-wide prediction of Polycomb/Trithorax response elements in *Drosophila melanogaster*. Dev Cell 5: 759–771. 14602076 10.1016/s1534-5807(03)00337-x

[ERCEGGAD292870C70] Schuettengruber B, Ganapathi M, Leblanc B, Portoso M, Jaschek R, Tolhuis B, van Lohuizen M, Tanay A, Cavalli G. 2009 Functional anatomy of polycomb and trithorax chromatin landscapes in *Drosophila* embryos. PLoS Biol 7: e13. 19143474 10.1371/journal.pbio.1000013 PMC2621266

[ERCEGGAD292870C71] Schuettengruber B, Martinez AM, Iovino N, Cavalli G. 2011 Trithorax group proteins: switching genes on and keeping them active. Nat Rev Mol Cell Biol 12: 799–814. 22108599 10.1038/nrm3230

[ERCEGGAD292870C72] Schuettengruber B, Oded Elkayam N, Sexton T, Entrevan M, Stern S, Thomas A, Yaffe E, Parrinello H, Tanay A, Cavalli G. 2014 Cooperativity, specificity, and evolutionary stability of Polycomb targeting in *Drosophila*. Cell Rep 9: 219–233. 25284790 10.1016/j.celrep.2014.08.072

[ERCEGGAD292870C73] Schwartz YB, Pirrotta V. 2013 A new world of Polycombs: unexpected partnerships and emerging functions. Nat Rev Genet 14: 853–864. 24217316 10.1038/nrg3603

[ERCEGGAD292870C74] Schwartz YB, Kahn TG, Nix DA, Li XY, Bourgon R, Biggin M, Pirrotta V. 2006 Genome-wide analysis of Polycomb targets in *Drosophila melanogaster*. Nat Genet 38: 700–705. 16732288 10.1038/ng1817

[ERCEGGAD292870C75] Sengupta AK, Kuhrs A, Muller J. 2004 General transcriptional silencing by a Polycomb response element in *Drosophila*. Development 131: 1959–1965. 15056613 10.1242/dev.01084

[ERCEGGAD292870C76] Simon JA, Kingston RE. 2009 Mechanisms of polycomb gene silencing: knowns and unknowns. Nat Rev Mol Cell Biol 10: 697–708. 19738629 10.1038/nrm2763

[ERCEGGAD292870C77] Simon JA, Kingston RE. 2013 Occupying chromatin: Polycomb mechanisms for getting to genomic targets, stopping transcriptional traffic, and staying put. Mol Cell 49: 808–824. 23473600 10.1016/j.molcel.2013.02.013 PMC3628831

[ERCEGGAD292870C78] Simon J, Chiang A, Bender W, Shimell MJ, O'Connor M. 1993 Elements of the *Drosophila* bithorax complex that mediate repression by Polycomb group products. Dev Biol 158: 131–144. 8101171 10.1006/dbio.1993.1174

[ERCEGGAD292870C79] Spitz F, Furlong EE. 2012 Transcription factors: from enhancer binding to developmental control. Nat Rev Genet 13: 613–626. 22868264 10.1038/nrg3207

[ERCEGGAD292870C80] Struhl G. 1981 A gene product required for correct initiation of segmental determination in *Drosophila*. Nature 293: 36–41. 7266657 10.1038/293036a0

[ERCEGGAD292870C81] Thomas S, Li XY, Sabo PJ, Sandstrom R, Thurman RE, Canfield TK, Giste E, Fisher W, Hammonds A, Celniker SE, 2011 Dynamic reprogramming of chromatin accessibility during *Drosophila* embryo development. Genome Biol 12: R43. 21569360 10.1186/gb-2011-12-5-r43 PMC3219966

[ERCEGGAD292870C82] Tolhuis B, de Wit E, Muijrers I, Teunissen H, Talhout W, van Steensel B, van Lohuizen M. 2006 Genome-wide profiling of PRC1 and PRC2 Polycomb chromatin binding in *Drosophila melanogaster*. Nat Genet 38: 694–699. 16628213 10.1038/ng1792

[ERCEGGAD292870C83] Von Ohlen T, Hooper JE. 1997 Hedgehog signaling regulates transcription through Gli/Ci binding sites in the wingless enhancer. Mech Dev 68: 149–156. 9431812 10.1016/s0925-4773(97)00150-0

[ERCEGGAD292870C84] Wang L, Brown JL, Cao R, Zhang Y, Kassis JA, Jones RS. 2004 Hierarchical recruitment of polycomb group silencing complexes. Mol Cell 14: 637–646. 15175158 10.1016/j.molcel.2004.05.009

[ERCEGGAD292870C85] Weiss A, Charbonnier E, Ellertsdottir E, Tsirigos A, Wolf C, Schuh R, Pyrowolakis G, Affolter M. 2010 A conserved activation element in BMP signaling during *Drosophila* development. Nat Struct Mol Biol 17: 69–76. 20010841 10.1038/nsmb.1715

[ERCEGGAD292870C86] Xiao J, Wagner D. 2015 Polycomb repression in the regulation of growth and development in *Arabidopsis*. Curr Opin Plant Biol 23: 15–24. 25449722 10.1016/j.pbi.2014.10.003

[ERCEGGAD292870C87] Zinzen RP, Girardot C, Gagneur J, Braun M, Furlong EE. 2009 Combinatorial binding predicts spatio–temporal *cis*-regulatory activity. Nature 462: 65–70. 19890324 10.1038/nature08531

