# Supplementary material for: Dual functionality of cis-regulatory elements as developmental enhancers and Polycomb response elements
Source: Genes Dev. 2017 Mar 15;31(6):590–602. doi: 10.1101/gad.292870.116 (PMC5393054; doi:10.1101/gad.292870.116)
Supplement: Supplemental Material [file supp_31_6_590__index.html]

Dual functionality of cis-regulatory elements as developmental enhancers and Polycomb response elements — Supplemental Material 

# Dual functionality of *cis*-regulatory elements as developmental enhancers and Polycomb response elements

## Supplemental Material

undefined

- Supplemental\_Figures\_Methods.docx
- Supplemental\_Table\_S3.xlsx
- Supplemental\_Table\_S1.xlsx
- Supplemental\_Table\_S2.xlsx
