# Supplementary material for: Dual functionality of cis-regulatory elements as developmental enhancers and Polycomb response elements
Source: Genes Dev. 2017 Mar 15;31(6):590–602. doi: 10.1101/gad.292870.116 (PMC5393054; doi:10.1101/gad.292870.116)
Supplement: Supplemental Material [file supp_gad.292870.116_Supplemental_Figs_Methods.pdf]

Supplementary Information

Dual functionality of *cis*-regulatory elements as developmental enhancers and Polycomb response elements

Jelena Erceg[*], Tibor Pakozdi[*], Raquel Marco-Ferreres[*], Yad Ghavi-Helm, Charles Girardot, Adrian P. Bracken, Eileen E.M. Furlong[†]

SUPPLEMENTARY FIGURES

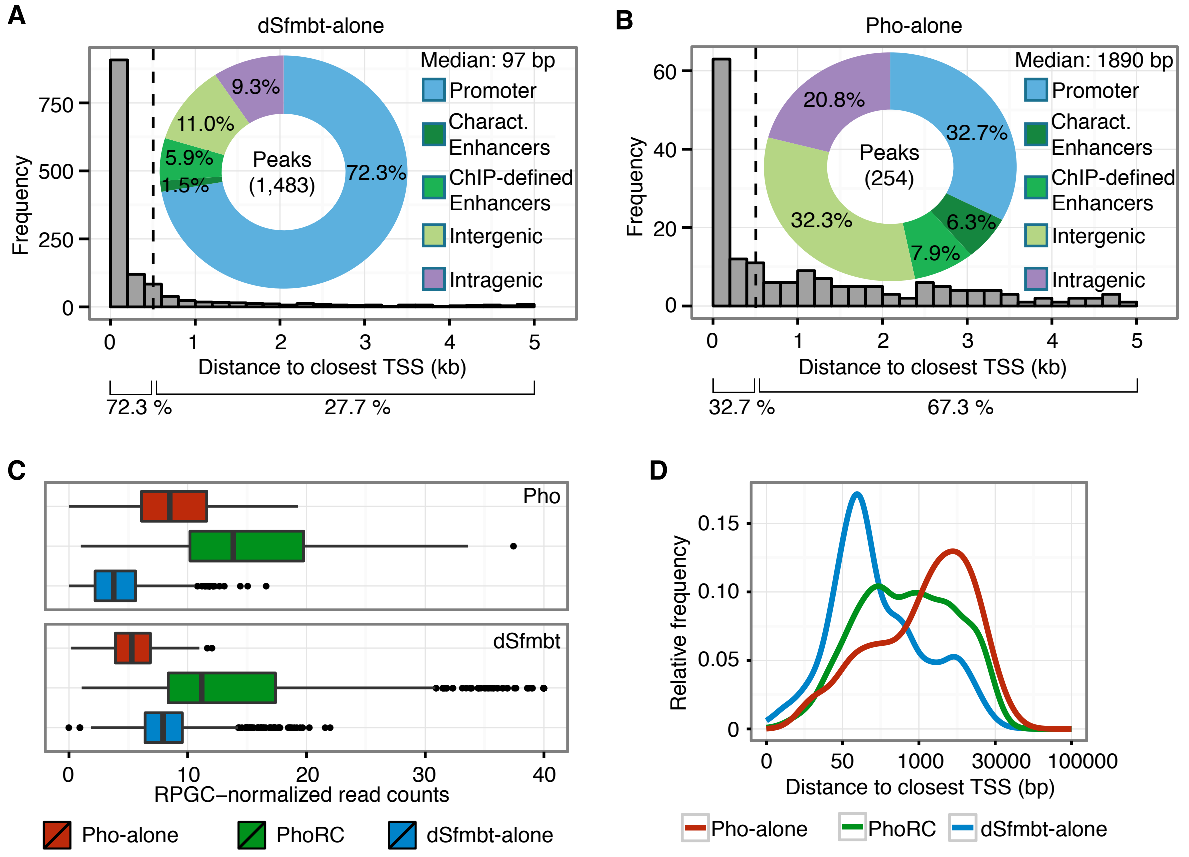

Figure S1: Distribution of regions bound by dSfmbt-only and Pho-only

(A,B) Frequency of dSfmbt-only **(A)** and Pho-only **(B)** ChIP peak summits relative to the distance from the closest TSS (histogram). The percentage of peaks (doughnut) over-lapping promoters, characterized enhancers, ChIP defined enhancers, intergenic, and intragenic regions (similar to Fig. 1a for PhoRC). **(C)** Quantitative ChIP signal (read counts) for Pho and dSfmbt at 6-8h at regions bound by PhoRC (green), Pho-alone (red) and dSfmbt-alone (blue). **(D)** Relative frequency of distances of PhoRC, Pho-alone and dSfmbt-alone peak summits to the closest TSS.

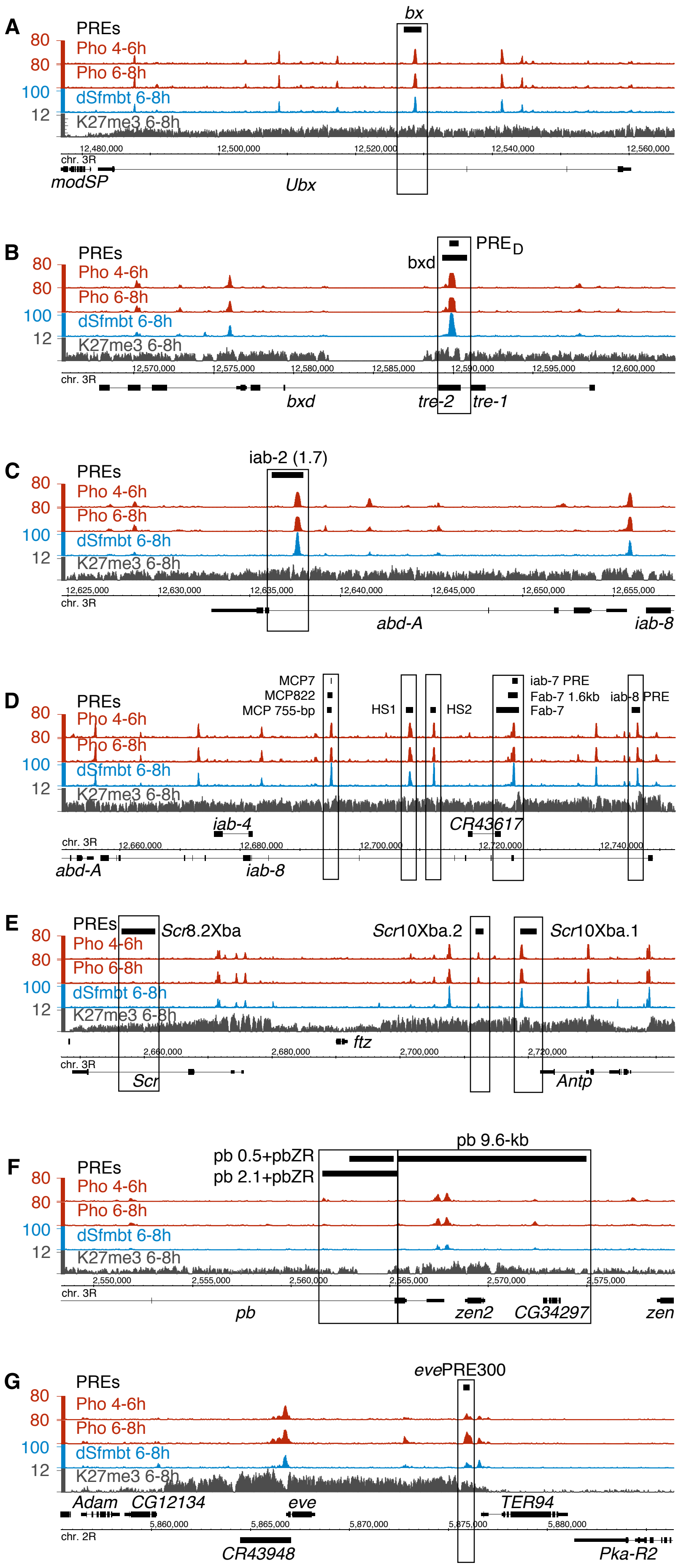
A
bx
PREs
Pho 4-6h
Pho 6-8h
dSfmbt 6-8h
K27me3 6-8h
80
80
100
12
12,480,000
12,500,000
12,520,000
12,540,000
12,560,000
chr. 3R
modSP
Ubx
B
PREs
bxd
PRE$_D$
Pho 4-6h
Pho 6-8h
dSfmbt 6-8h
K27me3 6-8h
12,570,000
12,575,000
12,580,000
12,585,000
12,590,000
12,595,000
12,600,000
chr. 3R
bxd
tre-2
tre-1
C
iab-2 (1.7)
PREs
Pho 4-6h
Pho 6-8h
dSfmbt 6-8h
K27me3 6-8h
12,625,000
12,630,000
12,635,000
12,640,000
12,645,000
12,650,000
12,655,000
chr. 3R
abd-A
iab-8
D
MCP7
MCP822
MCP 755-bp
HS1
HS2
iab-7 PRE
Fab-7 1.6kb
Fab-7
iab-8 PRE
PREs
Pho 4-6h
Pho 6-8h
dSfmbt 6-8h
K27me3 6-8h
iab-4
CR43617
chr. 3R
12,660,000
12,680,000
12,700,000
12,720,000
12,740,000
abd-A
iab-8
E
PREs
Scr8.2Xba
Scr10Xba.2
Scr10Xba.1
Pho 4-6h
Pho 6-8h
dSfmbt 6-8h
K27me3 6-8h
ftz
2,660,000
2,680,000
2,700,000
2,720,000
chr. 3R
Scr
Antp
F
pb 9.6-kb
pb 0.5+pbZR
pb 2.1+pbZR
PREs
Pho 4-6h
Pho 6-8h
dSfmbt 6-8h
K27me3 6-8h
2,550,000
2,555,000
2,560,000
2,565,000
2,570,000
2,575,000
chr. 3R
pb
zen2
CG34297
zen
G
evePRE300
PREs
Pho 4-6h
Pho 6-8h
dSfmbt 6-8h
K27me3 6-8h
Adam
CG12134
eve
TER94
5,860,000
5,865,000
5,870,000
5,875,000
5,880,000
chr. 2R
CR43948
Pka-R2

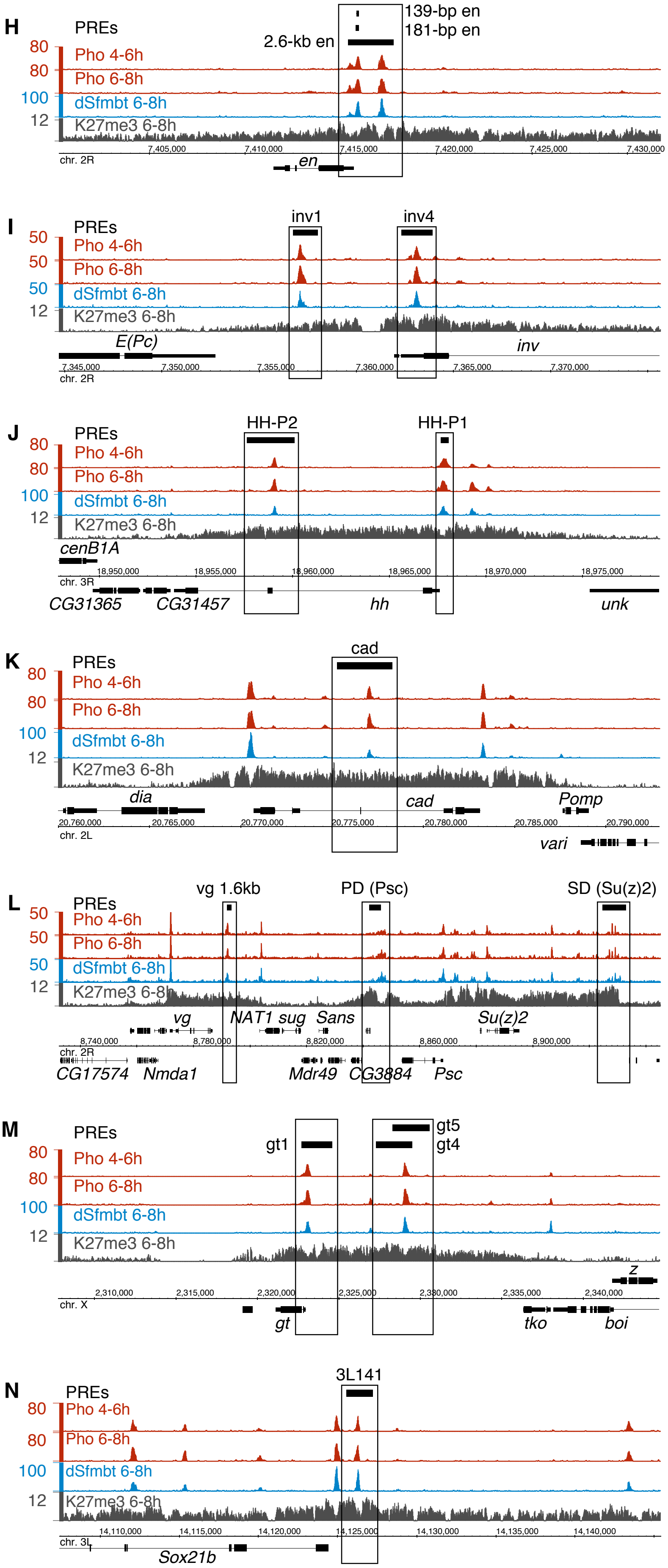
H
PREs
139-bp en
181-bp en
2.6-kb en
Pho 4-6h
Pho 6-8h
dSfmbt 6-8h
K27me3 6-8h
chr. 2R
en
I
inv1
inv4
E(Pc)
inv
J
HH-P2
HH-P1
cenB1A
CG31365
CG31457
hh
unk
chr. 3R
K
cad
dia
Pomp
vari
chr. 2L
L
vg 1.6kb
PD (Psc)
SD (Su(z)2)
vg
NAT1
sug
Sans
Su(z)2
CG17574
Nmda1
Mdr49
CG3884
Psc
M
gt1
gt5
gt4
z
gt
tko
boi
chr. X
N
3L141
Sox21b
chr. 3L

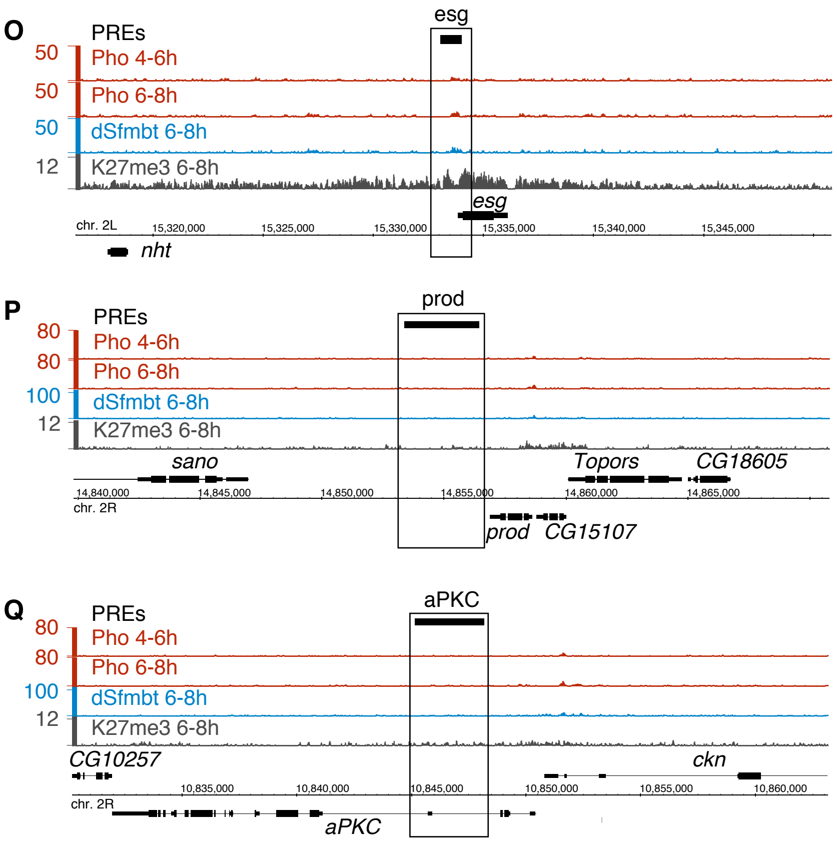

Figure S2: PhoRC occupancy at functionally characterized Polycomb response elements (PREs)

(A-Q) ChIP signal for Pho, dSfmbt (red, blue respectively; input subtracted), and histone modification H3K27me3 (grey, H3 subtracted) (Bonn et al. 2012a), characterized PREs indicated above (black) at **(A-D)** Bithorax (Karch et al. 1994; Hagstrom et al. 1997; Orlando et al. 1998; Fritsch et al. 1999; Barges et al. 2000; Shimell et al. 2000; Busturia et al. 2001; Gruzdeva et al. 2005; Perez-Lluch et al. 2008; Okulski et al. 2011), **(E,F)** Antennapedia (Gindhart and Kaufman 1995; Kapoun and Kaufman 1995; Ringrose et al. 2003) complexes, **(G)** *eve* (Fujioka et al. 2008), **(H)** *engrailed* (Americo et al. 2002), **(I)** *invected* (Cunningham et al. 2010), **(J)** *hedgehog* (Chanas and Maschat 2005), **(K)** *caudal* (Ringrose et al. 2003), **(L)** *vestigial* (Okulski et al. 2011), PcG genes *Psc* and *Su(z)2* (Park et al. 2012), **(M)** *gaint* (Abed et al. 2013), **(N)** *Sox21b* (Schuettengruber et al. 2014) **(O)** *escargot* (Kassis 1994), **(P)** *proliferation disrupter* (Ringrose et al. 2003), and **(Q)** *atypical Protein Kinase C* (Ringrose et al. 2003) loci. We observe binding at all previously characterized PREs (references indicated), with the exception of *Scr*8.2Xba, *prod* and *aPKC*. For *Scr*8.2Xba, the following studies also observed no PcG occupancy at this element (Kwong et al. 2008; Oktaba et al. 2008; Schuettengruber et al. 2009).

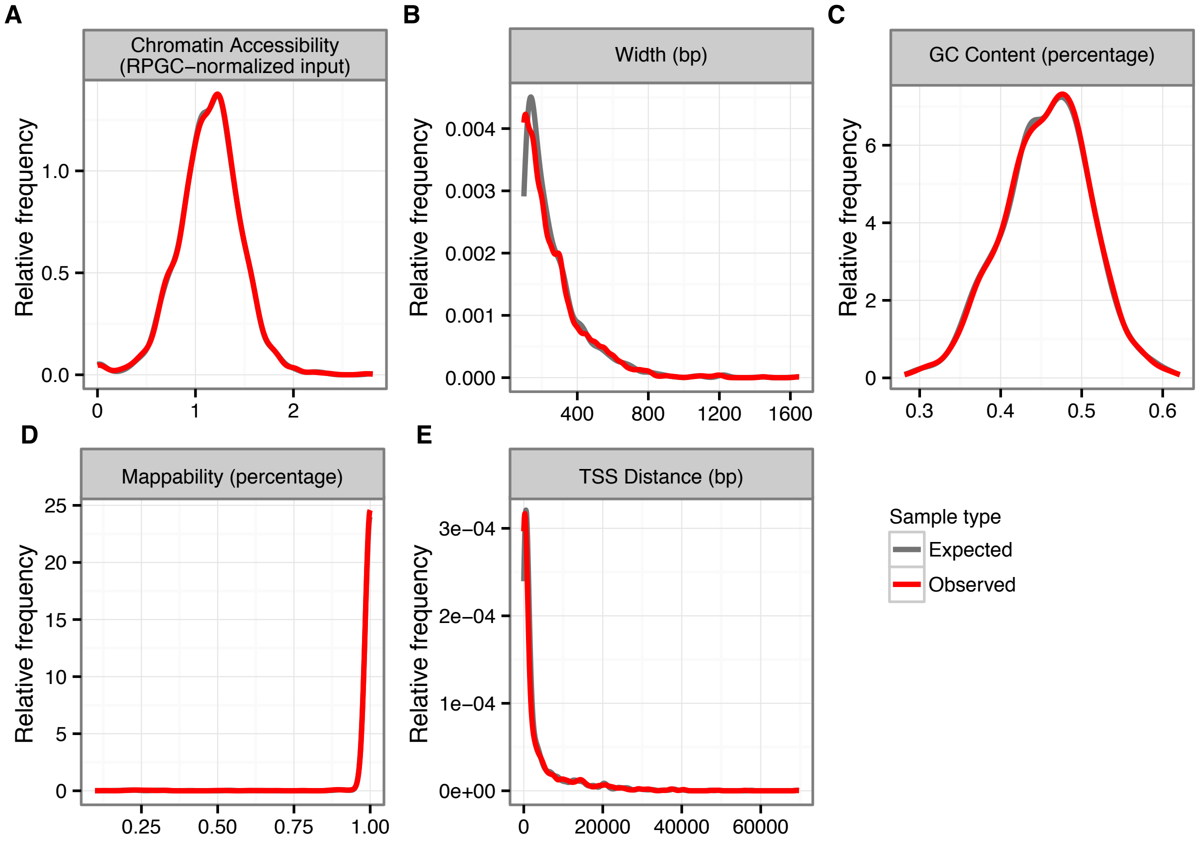

Figure S3: Matched background control regions with similar genomic properties as PhoRC peaks

Background regions were generated to match the PhoRC peaks in five properties: (**A**) chromatin accessibility, (**B**) width distribution, (**C**) GC dinucleotide content, (**D**) mappability, and (**E**) TSS-distance. The observed signals on the 994 genome-wide PhoRC loci is depicted in red, while the matched background set of equal size is in grey. As the properties are identical, the two plots are superimposed.

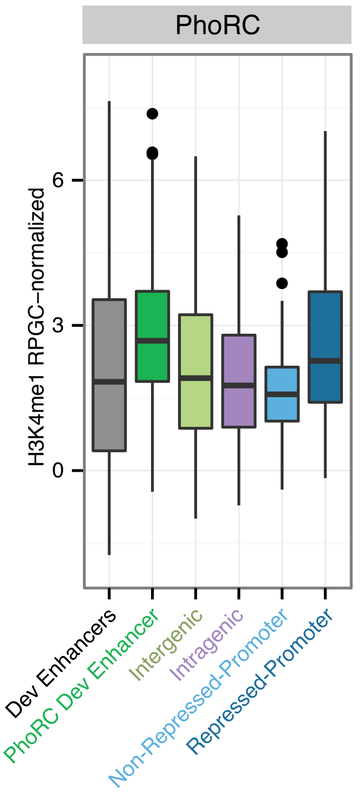

Figure S4: H3K4me1 signal at different genomic elements

Mesoderm-specific signal of H3K4me1 (Bonn et al. 2012a) is shown at PhoRC bound peaks categorized into six genomic regions: Developmental enhancers (grey), PhoRC-bound developmental enhancers (green), intergenic regions (light green), intragenic regions (purple), non-repressed-promoter (light blue), or repressed-promoter (dark blue). H3K4me1 signal is highest at PhoRC-bound developmental enhancers (dark green) and repressed promoters (dark blue), and also enriched at many PhoRC-bound intergenic regions (light green), as seen by the spread of the H3K4me1 distribution,

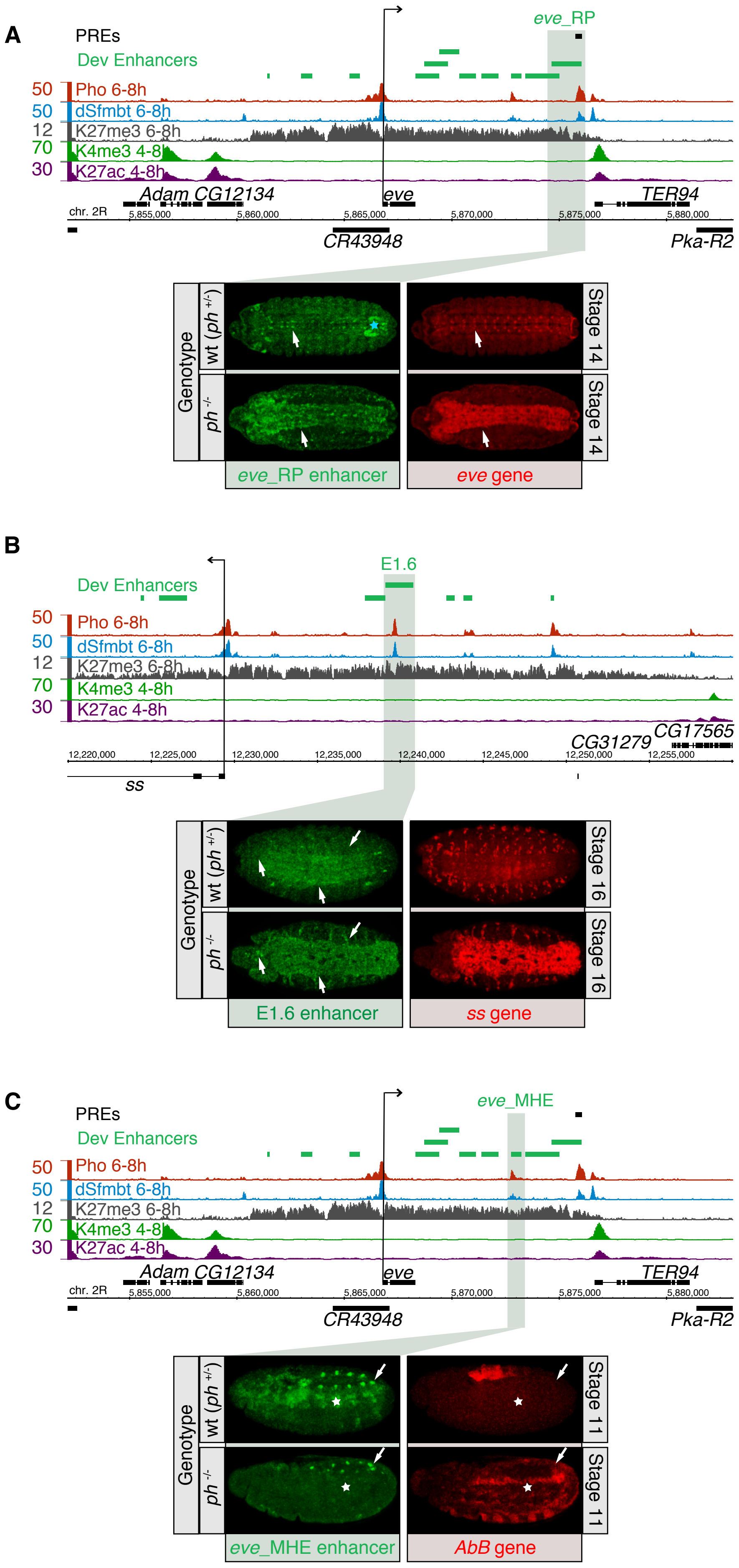

A
PREs
Dev Enhancers
eve_RP
Pho 6-8h
dSfmbt 6-8h
K27me3 6-8h
K4me3 4-8h
K27ac 4-8h
Adam CG12134
eve
TER94
chr. 2R
5,855,000
5,860,000
5,865,000
5,870,000
5,875,000
5,880,000
CR43948
Pka-R2
Genotype
wt (ph +/-)
ph -/-
Stage 14
eve_RP enhancer
eve gene
B
Dev Enhancers
E1.6
Pho 6-8h
dSfmbt 6-8h
K27me3 6-8h
K4me3 4-8h
K27ac 4-8h
CG31279
CG17565
12,220,000
12,225,000
12,230,000
12,235,000
12,240,000
12,245,000
12,250,000
12,255,000
ss
Stage 16
E1.6 enhancer
ss gene
C
PREs
Dev Enhancers
eve_MHE
Stage 11
eve_MHE enhancer
AbB gene

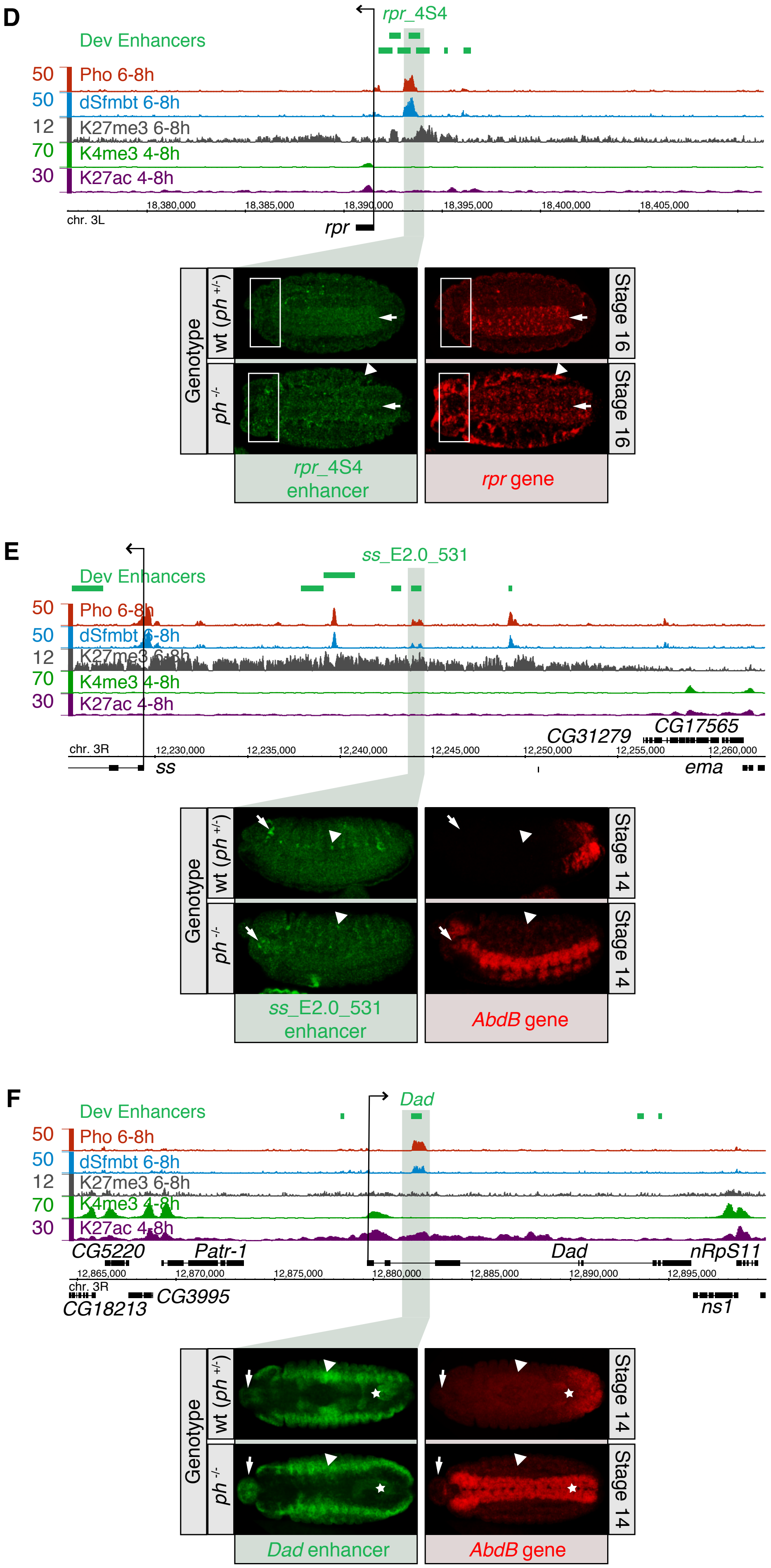
D
rpr_4S4
Dev Enhancers
50 Pho 6-8h
50 dSfmbt 6-8h
12 K27me3 6-8h
70 K4me3 4-8h
30 K27ac 4-8h
18,380,000
18,385,000
18,390,000
18,395,000
18,400,000
18,405,000
chr. 3L
rpr
Genotype
wt (ph +/-)
ph -/-
Stage 16
Stage 16
rpr_4S4 enhancer
rpr gene
E
ss_E2.0_531
Dev Enhancers
50 Pho 6-8h
50 dSfmbt 6-8h
12 K27me3 6-8h
70 K4me3 4-8h
30 K27ac 4-8h
CG31279
CG17565
chr. 3R
12,230,000
12,235,000
12,240,000
12,245,000
12,250,000
12,255,000
12,260,000
ss
ema
Genotype
wt (ph +/-)
ph -/-
Stage 14
Stage 14
ss_E2.0_531 enhancer
AbdB gene
F
Dad
Dev Enhancers
50 Pho 6-8h
50 dSfmbt 6-8h
12 K27me3 6-8h
70 K4me3 4-8h
30 K27ac 4-8h
CG5220
Patr-1
Dad
nRpS11
12,865,000
12,870,000
12,875,000
12,880,000
12,885,000
12,890,000
12,895,000
chr. 3R
CG3995
CG18213
ns1
Genotype
wt (ph +/-)
ph -/-
Stage 14
Stage 14
Dad enhancer
AbdB gene

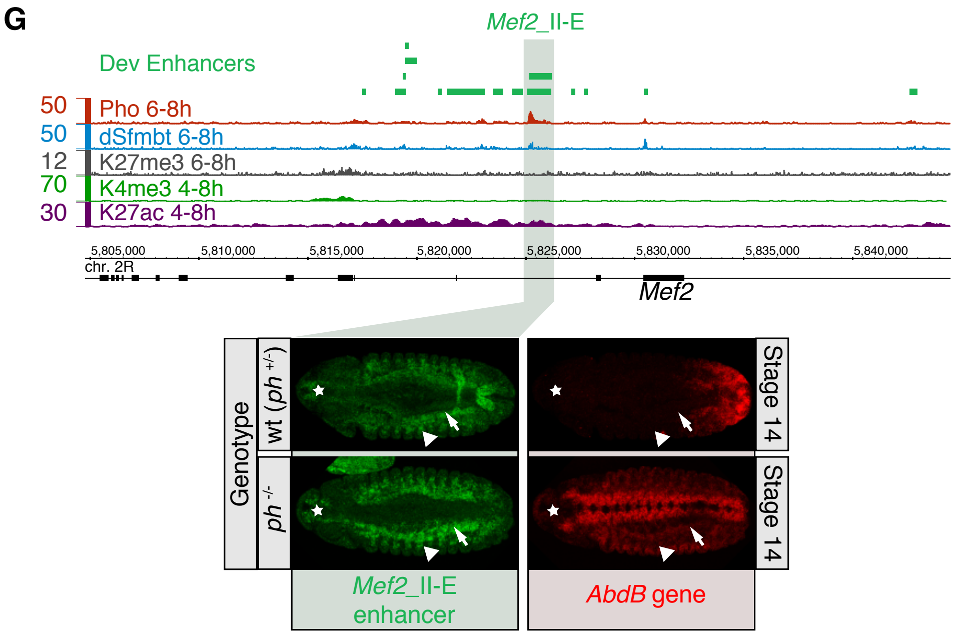

Figure S5: Assessing characterized developmental enhancers for PRE activity

(A-G) *Upper panels:* genomic locus showing ChIP-seq signal for Pho (red), dSfmbt (blue) (background subtracted) and H3K27me3 (H3 subtracted, (Bonn et al. 2012a) from mesodermal cells and H3K4me3, H3K27ac from whole-embryos (modENCODE, H3 subtracted). Characterized developmental enhancers (green) are indicated: **(A)** *eve*_RP (McDonald et al. 2003), **(B)** E1.6 (Emmons et al. 2007), **(C)** *eve*_MHE (Halfon et al. 2000; Knirr and Frasch 2001; Han et al. 2002), **(D)** *rpr*_4S4 (Lohmann 2003), **(E)** *ss*_E2.0_531 (Emmons et al. 2007), **(F)** *Dad* (Weiss et al. 2010), **(G)** *Mef2*_II-E (Nguyen and Xu 1998). *Lower panels: In situ* hybridization against the *mini-white* gene driven by the characterized developmental enhancer (green) and the associated endogenous gene (red) or a PcG responsive gene, *AbdB* (red), to distinguish the genetic background (Gambetta and Muller 2014) - heterozygous *ph*$^{+/-}$ and homozygous *ph*$^{-/-}$ mutant embryos. **(A, B)** Reporter gene expression driven by the *eve*_RP and E1.6 enhancers are derepressed in *ph*$^{-/-}$ background in neurons (arrow). **(C)** Expression of enhancer *eve*_MHE is substantially reduced in the midgut visceral mesoderm (asterisk), but not in the pericardial and muscle precursors (arrow), with no obvious derepression. PcG therefore seems to have a positive effect (either directly or indirectly) on enhancer midgut activity. **(D-F)** Three enhancers with possible weak depression in the anterior head region. **(D)** The *rpr*_4S4 enhancer appears derepressed in the head region (white box) and PNS (arrowhead) in *ph*$^{-/-}$ mutant, the *rpr* gene is

upregulated in the central nervous system (CNS, arrow). **(E,F)** The activity of *ss*_E2.0_531 and *Dad* enhancers are largely unaltered in the $ph^{-/-}$ mutant, with perhaps some weak misexpression in the head region. The *ss*_E2.0_531 enhancer is active in the peripheral nervous system (arrowhead), and eye antennal disc (arrow) **(E)**, *Dad* enhancer in the anterior head structures (arrowhead), two ectodermal stripes (arrow), and gut (asterisk). Although these 3 enhancers might be influenced by PcG, given the weak and variable anterior staining in *ph* mutants, we have not considered them as depressed enhancers. **(G)** *Mef2*_II-E enhancer, active in pharyngeal (asterisk), longitudinal (arrowhead) and somatic muscles (arrow), is unaltered in $ph^{-/-}$ mutant. Blue asterisk (in A) depicts background staining of endogenous *white* gene (Fjose et al. 1984). Embryos are ventrally **(A, B, D, F, G)** or laterally **(C, E)** oriented with anterior to the left.

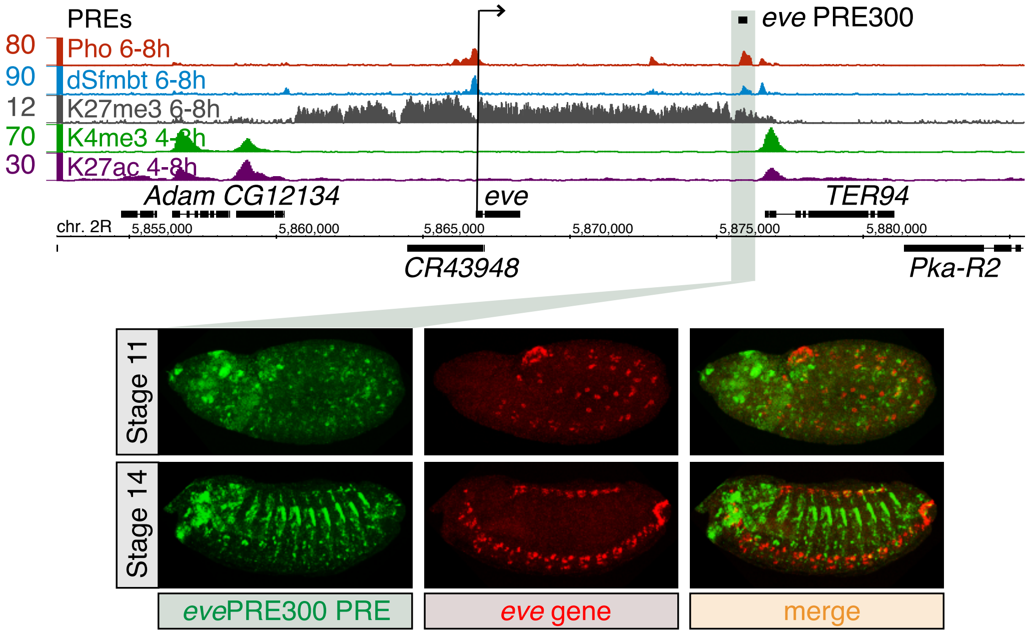

Figure S6: Assessing characterized PREs for developmental enhancer activity

Upper panel: genomic locus showing ChIP-seq signal (background subtracted) for Pho (red), dSfmbt (blue) and H3K27me3 (Bonn et al. 2012a) from mesodermal cells and whole-embryo ChIP-seq signal for H3K4me3 and H3K27ac (modENCODE, H3 subtracted). Characterized *eve*PRE300 (Fujioka et al. 2008) (black) is indicated. *Lower panel: In situ* hybridization against the *lacZ* reporter gene driven by the characterized PRE (green), and the associated endogenous gene (red) at two stages of development. Embryos are laterally oriented with anterior to the left. In addition to the *eve*PRE300, and the three PREs presented in Fig. 5, we also tested the PRE_D and MCP822 PREs. The PRE_D (Fritsch et al. 1999) gave background activity in a pattern similar to the empty vector, while the MCP822 PRE (Busturia et al. 2001) had no staining (data not shown), both therefore cannot function as enhancers in this context.

SUPPLEMENTARY METHODS

PhoRC BiTS-ChIP-Seq

Whole embryos from a transgenic line containing a mesodermal driven tagged histone H2B (twist: SBP-H2B) (Bonn et al. 2012a) were collected and fixed at 4-6h (spanning stages 8-9) and 6-8h (stages 10-11) of embryogenesis and used to perform mesoderm-specific ChIP as previously described in the detailed Batch isolate Tissue-specific Chromatin for Immunoprecipitation (BiTS-ChIP) protocol (Bonn et al. 2012b). Briefly, formaldehyde fixed whole embryos were homogenized and dissociated by pipetting through needles to extract intact separated nuclei. Nuclei were stained with a mouse anti- α-SBP (Streptavidin Binding Protein) antibody and a α-mouse Alexa Fluor 488 secondary antibody to stain mesodermal nuclei, which were then separated using Fluorescence Activated Cell Sorting (FACS) to isolate mesodermal nuclei with a purity >95%. For some samples, several sorts were pulled together to obtain sufficient amount of material. Chromatin was sheared to 200 bp with a Bioruptor and used to perform imunoprecipitation (IP) as previously described (Sandmann et al. 2006) with characterized antibodies (a generous gifts from Jürg Müller (Klymenko et al. 2006)) recognizing Pho (2-382 aa) or dSfmbt (531-980 aa). ChIP conditions were optimized using ChIP-qPCR with positive and negative controls to obtain the optimal balance between good recovery and enrichment. Here, 10 μg of chromatin was used to obtain 2-3 ng of IP-ed material to generate Solexa libraries with 18 cycles of PCR amplification. For each time point, two independent biological replicates were generated for each antibody and sequenced on either Illumina GA_IIx (Pho) or Hi-Seq machines (dSfmbt) by the EMBL Genomics Core facility.

ChIP-qPCR of H3K27me3 from transgenic enhancer lines

Embryos were collected from five transgenic enhancer lines and the 'landing site' line where each of the enhancers were inserted (no enhancer), at 4-16hrs of development. The landing site line 16a (in band 46E1) is from Okulski *et al* (Okulski et al. 2011) and carries an attP site (to allow all enhancers to be inserted into the same genomic location) and approximately half of the mini-white gene (pKC27). All embryos were formaldehyde fixed and used for chromatin preparations as described previously (Sandmann et al. 2006). ChIP was performed in two independent biological replicates with ~10µg of chromatin and 3 µl of H3K27me3 Abcam antibody (ab6002) per ChIP. ChIP-qPCR was performed using positive (designed to amplify from the integrated transgene enhancer sequence (primers are labeled by the name of the enhancer) or the landing site (pCK27)) and negative primers, with the following sequences:

wg-L GAACTCTGAATAGGGAATTGGGA
wg-R TTTTACGAAATGCCTGCCTTAAT
ey/eveRP-L ACTGCACTGGATATCATTGAACT
ey/eveRP-R ACATCAAATACCCTTGGATCGA
Ubx-L TTCGTTAACAGATCTGCGGC
Ubx-R TTTTACCCGGCTTTCAACCC
E1.6-L ATTCGTTAACAGATCTGCGGC
E1.6-R AAGTAAACTACCTCCTCGAGCC
pKC27-R CGGTGATGACGGTGAAAACC
pKC27_L AGACAAGCTGTGACCGTCTC
Negative primers: RPL32N-F GGCACGGCGCCAAAATTAATCA
RPL32N-R ccgatgccactgcctctttggt

ChIP-Seq data processing

To make the dSfmbt data, which was sequenced as 50bp single end reads, more comparable to Pho (sequenced on an Illumina GA_IIx as 36bp single end) reads, the FASTQ files for both biological replicates were trimmed to 36bp – matching the trimmed length of the sequenced Pho reads. All reads were aligned to the *Drosophila melanogaster* genome version 3 (July 2006; (Celniker and Rubin 2003)) using BWA v0.7.5a (Li and Durbin 2009), allowing for two mismatches and no gaps (-n 2 -o 0). Additionally '-I' parameter was used for Pho samples that contained Phred+64 quality encoding. Only non-duplicate uniquely aligned reads with the 'XT:A:U' tag were kept for further analysis. Reads aligned to unassembled contigs (U/Uextra) and the mitochondrial genome (M) were discarded. ChIP-seq forward and reverse strands read were shifted, as previously described (Park 2009). For all subsequent analysis, biological replicates were merged into single alignment files for each developmental stage and antibody using samtools v0.1.19-44428 (Li et al. 2009).

Peak calling

cisGenome v2.0 (Ji et al. 2008) was used to locate the enriched ChIP regions from two biological replicates compared to 4-6h and 6-8h input controls (input), using default parameters, with the exception of extending shifted reads by 36bp (-e 36), setting a higher neighboring peak threshold (-maxgap 200), and defining a stringent standardized t-statistic cutoff (-c 3.5). A union of Pho peaks at the two different developmental stages was taken to remove redundancy, followed by the intersection with dSfmbt peaks to define the PhoRC loci. Flybase annotation v5.9 (St Pierre et al. 2014) was used throughout the analysis in this study.

Normalization and visualization

Difference in sequencing depth between the libraries was corrected by using Reads Per Genome Coverage (RPGC) normalization (Bonn et al. 2012a), in which the total read count coverage was multiplied by the ratio of read length (36bp) and mappable genome size (1.35e+08). Corrected coverage was summarized into 20bp bins. For visualization tracks, ChIP samples were additionally subtracted with the appropriate input control.

Distal developmental enhancers

The list of developmental enhancers was constructed using (a) characterized enhancers from transgenic embryos ((Gallo et al. 2011; Bonn et al. 2012a; Kvon et al. 2014)), (b) ChIP-defined putative enhancers representing 8008 mesodermal enhancers based on the binding of five transcription factors (Zinzen et al. 2009), and 4041 enhancers bound by five TFs essential for cardiac development (Junion et al. 2012). Several steps were taken to remove redundancy between the datasets: 8008 enhancers that overlapped with characterized enhancers were removed, together with the cardiac enhancers that overlapped with the 8008 set, resulting in the unique set of 9,513 characterized and putative developmental enhancers. To focus on distal regulatory regions, we also removed all enhancers within 500bp of an annotated TSS (leaving 6,606 elements) and those that overlapped a H3K4me3 peak at 6-8h, to remove unannotated TSS, leaving a final set of 5,949 distal enhancers.

Construction of the background regions

To evaluate the significance of Pho colocalization on the defined set of developmental enhancers, a background set of regions was constructed by randomly sampling 124,800 starting positions over the *Drosophila melanogaster* genome, followed by a calculation of the following parameters for each region to find random elements with similar general properties

(Fig. S3): mappability (defined as percentage of mapped reads per base pair), local GC content, region width, chromatin accessibility (defined as number of RPGC-normalized input reads) and TSS distance for both observed (1,248 peaks) and expected regions. A sampling algorithm from the R package MatchIt was used (Ho et al. 2011) with mahalanobis distance to find an equal number of expected regions, which most closely matched in their genomic properties to the observed set. Significance of enhancer occupancy by Pho to the observed versus expected set was calculated using Fisher's Exact Test.

Motif discovery

De novo motif discovery was performed on the feature-separated *Drosophila melanogaster* genome, version 3, 100bp +/- around the Pho peak summit using MEME v.4.9.1.1 (Bailey et al. 2009), with the following parameters: '-dna -oc promoter -nostatus -maxsize 1000000 -mod zoops -nmotifs 20 -minw 5 -maxw 50 -revcomp seq.fa'.

RNA-Seq

Mesoderm-specific RNA-Seq data (Gaertner et al. 2012), from embryos at the same developmental stages as our ChIP experiments, was used to assess levels of gene expression (RPKM values). Genes were categorized into different classes based on their spatial expression using *in situ* hybridization data, as follows: 'Ubiq' (ubiquitously expressed), 'Meso' (genes expressed in mesoderm and potentially other tissues, but not ubiquitously), and 'Non-meso' (expression that lacks mesodermal annotation, but is not ubiquitous). In addition, two classes of enhancers were inspected: 'TF bound enhancer' having two or more associated mesodermal TFs (meso-TFs), and 'Non-bound enhancer' having no meso-TF occupancy, for the TFs with available ChIP data at 6-8h of embryogenesis. These enhancer classes were associated with the closest upstream or downstream gene, using a simple nearest neighbor gene assignment.

Testing if developmental enhancers can function as PREs in vivo

Endogenous enhancers for *Dad* (chr3R:12,881,893-12,882,568) (Weiss et al. 2010), E1.6 (chr3R:12,239,098-12,240,917) (Emmons et al. 2007), *eve*_MHE (chr2R:5,872,764-5,873,339) (Halfon et al. 2000; Knirr and Frasch 2001; Han et al. 2002), *eve*_RP (chr2R:5,874,659-5,876,104) (McDonald et al. 2003), *ey*_UE0.9 (chr4:724,592-725,357) (Adachi et al. 2003), *Mef2*_II-E (chr2R:5,825,058-5,826,232) (Nguyen and Xu 1998), *rpr*_4S4 (chr3L:18,393,267-18,393,972) (Lohmann 2003), *ss*_E2.0_531 (chr3R:12,243,818-12,244,456) (Emmons et al. 2007), *Ubx*_BXD-C (chr3R:12,575,844-12,576,318; (Christen and Bienz 1992)), and *wg*_del-wg (chr2L:7,302,243-7,303,449) (Von Ohlen and Hooper 1997) were amplified by PCR using genomic DNA from *Drosophila* wild-type embryos as a template. The amplified fragments were cloned into a split mini-white vector (pKC27_*mw* vector; (Okulski et al. 2011) to assess pairing sensitive silencing (PSS) using XhoI-XbaI restriction enzyme sites, except *ey*_UE0.9, which was cloned using HincII-XhoI, and *wg*-_del-wg using HincII-XbaI. All enhancers were verified by Sanger sequencing.

Transgenic flies were obtained by co-injection of the pKC27_*mw* constructs with the helper plasmid pKC40 encoding ΦC31 integrase in the mapped attP landing site 2 in (Okulski et al. 2011) (Cytological location chr2R, 46E1 genomic position 5,965,083). Newly eclosed homozygous and heterozygous siblings (still with meconium) were placed into a new vial and aged for 4 days. The eye color of these age-matched heterozygous and homozygous sibling were compared at day 4 to assess PSS. Eye pictures were taken under a SZX16 Olympus stereomicroscope at 100x magnification with a Spot Insight Camera using the VisiView Software (Visitron Systems).

To demonstrate silencing in a PcG dependent manner, transgenic flies containing the homozygous enhancers were crossed to a characterized *ph* loss-of-function mutant background, using the ph^{del} strain (*w* ph^{del} FRT19A / FM7C *twi*::EGFP), in which all exons

of *ph-d* and *ph-p* are deleted, except the first exon of *ph-p* that codes for only 12 amino acids (Parks et al. 2004; Feng et al. 2011).

Testing if characterized PREs can function as developmental enhancers in vivo

Endogenous PREs for MCP822 (chr3R:12,694,616-12,695,452; (Busturia et al. 2001)), PRE_D (chr3R:12,589,768-12,590,340; (Fritsch et al. 1999)), *bx* (chr3R:12,527,152-12,529,708; (Orlando et al. 1998)), *Scr*Xba.1 (chr3R:2,718,866-2,721,381; (Gindhart and Kaufman 1995; Ringrose et al. 2003)), P{C4-418bis} (chrX:2,030,445-2,033,298; (Bloyer et al. 2003)) and *eve*PRE300 (chr2R:5,875,769-5,876,078; (Fujioka et al. 2008)) were amplified by PCR using genomic DNA from *Drosophila* wild-type embryos as template. The amplified fragments were cloned into pH-lacZ-attB vector (a standard enhancer-reporter vector) using AscI-XhoI restriction enzyme sites, except for *bx* and *Scr*Xba.1, which were cloned using AscI-KpnI sites. All PRE sequences were verified by Sanger sequencing. Cloned PREs in pH-lacZ-attB vector were used to generate stable homozygous transgenic *lacZ*-reporter fly lines with phiC31 mediated site-specific integration in the mapped attP landing site of J27 fly line (chromosomal position on 2R-51C, (Bischof et al. 2007)). Enhancer activity was assayed by *in situ* hybridization against the *lacZ* reporter.

In situ hybridization of Drosophila embryos

Double fluorescent *in situ* hybridization was performed using standard a protocol as described previously (Furlong et al. 2001). The following ESTs or full length cDNAs from *Drosophila* Gene Collection (DGC) were used to generate labeled probes: RE43738 (*Ubx*), RE02607 (*wg*), AT29177 (*ss*), GH01157 (*ey*), GH08934 (*ph-p*), and RE47096 (*AbdB*). cDNAs for probes against *white, Mef2*, *rpr*, and *scr* were generous gifts from Haini N. Cai, M. Taylor, I. Lohmann, and U. Elling, respectively. *Dfd* (R. Zinzen) and *eve* were cloned

after PCR amplification. Images were taken using Zeiss LSM 510 META and LSM780 confocal microscopes.

SUPPLEMENTARY REFERENCES

Abed JA, Cheng CL, Crowell CR, Madigan LL, Onwuegbuchu E, Desai S, Benes J, Jones RS. 2013. Mapping polycomb response elements at the Drosophilla melanogaster giant locus. *G3 (Bethesda)* **3**: 2297-2304.

Adachi Y, Hauck B, Clements J, Kawauchi H, Kurusu M, Totani Y, Kang YY, Eggert T, Walldorf U, Furukubo-Tokunaga K et al. 2003. Conserved cis-regulatory modules mediate complex neural expression patterns of the eyeless gene in the Drosophila brain. *Mech Dev* **120**: 1113-1126.

Americo J, Whiteley M, Brown JL, Fujioka M, Jaynes JB, Kassis JA. 2002. A complex array of DNA-binding proteins required for pairing-sensitive silencing by a polycomb group response element from the Drosophila engrailed gene. *Genetics* **160**: 1561-1571.

Bailey TL, Boden M, Buske FA, Frith M, Grant CE, Clementi L, Ren J, Li WW, Noble WS. 2009. MEME SUITE: tools for motif discovery and searching. *Nucleic Acids Res* **37**: W202-208.

Barges S, Mihaly J, Galloni M, Hagstrom K, Muller M, Shanower G, Schedl P, Gyurkovics H, Karch F. 2000. The Fab-8 boundary defines the distal limit of the bithorax complex iab-7 domain and insulates iab-7 from initiation elements and a PRE in the adjacent iab-8 domain. *Development* **127**: 779-790.

Bischof J, Maeda RK, Hediger M, Karch F, Basler K. 2007. An optimized transgenesis system for Drosophila using germ-line-specific phiC31 integrases. *Proc Natl Acad Sci U S A* **104**: 3312-3317.

Bloyer S, Cavalli G, Brock HW, Dura JM. 2003. Identification and characterization of polyhomeotic PREs and TREs. *Dev Biol* **261**: 426-442.

Bonn S, Zinzen RP, Girardot C, Gustafson EH, Perez-Gonzalez A, Delhomme N, Ghavi-Helm Y, Wilczynski B, Riddell A, Furlong EE. 2012a. Tissue-specific analysis of chromatin state identifies temporal signatures of enhancer activity during embryonic development. *Nat Genet* **44**: 148-156.

Bonn S, Zinzen RP, Perez-Gonzalez A, Riddell A, Gavin AC, Furlong EE. 2012b. Cell type-specific chromatin immunoprecipitation from multicellular complex samples using BiTS-ChIP. *Nat Protoc* **7**: 978-994.

Busturia A, Lloyd A, Bejarano F, Zavortink M, Xin H, Sakonju S. 2001. The MCP silencer of the Drosophila Abd-B gene requires both Pleiohomeotic and GAGA factor for the maintenance of repression. *Development* **128**: 2163-2173.

Celniker SE, Rubin GM. 2003. The Drosophila melanogaster genome. *Annu Rev Genomics Hum Genet* **4**: 89-117.

Chanas G, Maschat F. 2005. Tissue specificity of hedgehog repression by the Polycomb group during Drosophila melanogaster development. *Mech Dev* **122**: 975-987.

Christen B, Bienz M. 1992. A cis-element mediating Ultrabithorax autoregulation in the central nervous system. *Mech Dev* **39**: 73-80.

Cunningham MD, Brown JL, Kassis JA. 2010. Characterization of the polycomb group response elements of the Drosophila melanogaster invected Locus. *Mol Cell Biol* **30**: 820-828.

Emmons RB, Duncan D, Duncan I. 2007. Regulation of the Drosophila distal antennal determinant spineless. *Dev Biol* **302**: 412-426.

Feng S, Huang J, Wang J. 2011. Loss of the Polycomb group gene polyhomeotic induces non-autonomous cell overproliferation. *EMBO Rep* **12**: 157-163.

Fjose A, Polito LC, Weber U, Gehring WJ. 1984. Developmental expression of the white locus of Drosophila melanogaster. *EMBO J* **3**: 2087-2094.

Fritsch C, Brown JL, Kassis JA, Muller J. 1999. The DNA-binding polycomb group protein pleiohomeotic mediates silencing of a Drosophila homeotic gene. *Development* **126**: 3905-3913.

Fujioka M, Yusibova GL, Zhou J, Jaynes JB. 2008. The DNA-binding Polycomb-group protein Pleiohomeotic maintains both active and repressed transcriptional states through a single site. *Development* **135**: 4131-4139.

Furlong EE, Andersen EC, Null B, White KP, Scott MP. 2001. Patterns of gene expression during Drosophila mesoderm development. *Science* **293**: 1629-1633.

Gaertner B, Johnston J, Chen K, Wallaschek N, Paulson A, Garruss AS, Gaudenz K, De Kumar B, Krumlauf R, Zeitlinger J. 2012. Poised RNA polymerase II changes over developmental time and prepares genes for future expression. *Cell Rep* **2**: 1670-1683.

Gallo SM, Gerrard DT, Miner D, Simich M, Des Soye B, Bergman CM, Halfon MS. 2011. REDfly v3.0: toward a comprehensive database of transcriptional regulatory elements in Drosophila. *Nucleic Acids Res* **39**: D118-123.

Gambetta MC, Muller J. 2014. O-GlcNAcylation prevents aggregation of the Polycomb group repressor polyhomeotic. *Dev Cell* **31**: 629-639.

Gindhart JG, Jr., Kaufman TC. 1995. Identification of Polycomb and trithorax group responsive elements in the regulatory region of the Drosophila homeotic gene Sex combs reduced. *Genetics* **139**: 797-814.

Gruzdeva N, Kyrchanova O, Parshikov A, Kullyev A, Georgiev P. 2005. The Mcp element from the bithorax complex contains an insulator that is capable of pairwise interactions and can facilitate enhancer-promoter communication. *Mol Cell Biol* **25**: 3682-3689.

Hagstrom K, Muller M, Schedl P. 1997. A Polycomb and GAGA dependent silencer adjoins the Fab-7 boundary in the Drosophila bithorax complex. *Genetics* **146**: 1365-1380.

Halfon MS, Carmena A, Gisselbrecht S, Sackerson CM, Jimenez F, Baylies MK, Michelson AM. 2000. Ras pathway specificity is determined by the integration of multiple signal-activated and tissue-restricted transcription factors. *Cell* **103**: 63-74.

Han Z, Fujioka M, Su M, Liu M, Jaynes JB, Bodmer R. 2002. Transcriptional integration of competence modulated by mutual repression generates cell-type specificity within the cardiogenic mesoderm. *Dev Biol* **252**: 225-240.

Ho D, Imai K, King G, Stuart EA. 2011. MatchIt: Nonparametric Preprocessing for Parametric Causal Inference. *Journal of Statistical Software* **42**: 1-28.

Ji H, Jiang H, Ma W, Johnson DS, Myers RM, Wong WH. 2008. An integrated software system for analyzing ChIP-chip and ChIP-seq data. *Nat Biotechnol* **26**: 1293-1300.

Junion G, Spivakov M, Girardot C, Braun M, Gustafson EH, Birney E, Furlong EE. 2012. A transcription factor collective defines cardiac cell fate and reflects lineage history. *Cell* **148**: 473-486.

Kapoun AM, Kaufman TC. 1995. Regulatory regions of the homeotic gene proboscipedia are sensitive to chromosomal pairing. *Genetics* **140**: 643-658.

Karch F, Galloni M, Sipos L, Gausz J, Gyurkovics H, Schedl P. 1994. Mcp and Fab-7: molecular analysis of putative boundaries of cis-regulatory domains in the bithorax complex of Drosophila melanogaster. *Nucleic Acids Res* **22**: 3138-3146.

Kassis JA. 1994. Unusual properties of regulatory DNA from the Drosophila engrailed gene: three "pairing-sensitive" sites within a 1.6-kb region. *Genetics* **136**: 1025-1038.

Klymenko T, Papp B, Fischle W, Kocher T, Schelder M, Fritsch C, Wild B, Wilm M, Muller J. 2006. A Polycomb group protein complex with sequence-specific DNA-binding and selective methyl-lysine-binding activities. *Genes Dev* **20**: 1110-1122.

Knirr S, Frasch M. 2001. Molecular integration of inductive and mesoderm-intrinsic inputs governs even-skipped enhancer activity in a subset of pericardial and dorsal muscle progenitors. *Dev Biol* **238**: 13-26.

Kvon EZ, Kazmar T, Stampfel G, Yanez-Cuna JO, Pagani M, Schernhuber K, Dickson BJ, Stark A. 2014. Genome-scale functional characterization of Drosophila developmental enhancers in vivo. *Nature* **512**: 91-95.

Kwong C, Adryan B, Bell I, Meadows L, Russell S, Manak JR, White R. 2008. Stability and dynamics of polycomb target sites in Drosophila development. *PLoS Genet* **4**: e1000178.

Li H, Durbin R. 2009. Fast and accurate short read alignment with Burrows-Wheeler transform. *Bioinformatics* **25**: 1754-1760.

Li H, Handsaker B, Wysoker A, Fennell T, Ruan J, Homer N, Marth G, Abecasis G, Durbin R. 2009. The Sequence Alignment/Map format and SAMtools. *Bioinformatics* **25**: 2078-2079.

Lohmann I. 2003. Dissecting the regulation of the Drosophila cell death activator reaper. *Gene Expr Patterns* **3**: 159-163.

McDonald JA, Fujioka M, Odden JP, Jaynes JB, Doe CQ. 2003. Specification of motoneuron fate in Drosophila: integration of positive and negative transcription factor inputs by a minimal eve enhancer. *J Neurobiol* **57**: 193-203.

Nguyen HT, Xu X. 1998. Drosophila mef2 expression during mesoderm development is controlled by a complex array of cis-acting regulatory modules. *Dev Biol* **204**: 550-566.

Oktaba K, Gutierrez L, Gagneur J, Girardot C, Sengupta AK, Furlong EE, Muller J. 2008. Dynamic regulation by polycomb group protein complexes controls pattern formation and the cell cycle in Drosophila. *Dev Cell* **15**: 877-889.

Okulski H, Druck B, Bhalerao S, Ringrose L. 2011. Quantitative analysis of polycomb response elements (PREs) at identical genomic locations distinguishes contributions of PRE sequence and genomic environment. *Epigenetics Chromatin* **4**: 4.

Orlando V, Jane EP, Chinwalla V, Harte PJ, Paro R. 1998. Binding of trithorax and Polycomb proteins to the bithorax complex: dynamic changes during early Drosophila embryogenesis. *EMBO J* **17**: 5141-5150.

Park PJ. 2009. ChIP-seq: advantages and challenges of a maturing technology. *Nat Rev Genet* **10**: 669-680.

Park SY, Schwartz YB, Kahn TG, Asker D, Pirrotta V. 2012. Regulation of Polycomb group genes Psc and Su(z)2 in Drosophila melanogaster. *Mech Dev* **128**: 536-547.

Parks AL, Cook KR, Belvin M, Dompe NA, Fawcett R, Huppert K, Tan LR, Winter CG, Bogart KP, Deal JE et al. 2004. Systematic generation of high-resolution deletion coverage of the Drosophila melanogaster genome. *Nat Genet* **36**: 288-292.

Perez-Lluch S, Cuartero S, Azorin F, Espinas ML. 2008. Characterization of new regulatory elements within the Drosophila bithorax complex. *Nucleic Acids Res* **36**: 6926-6933.

Ringrose L, Rehmsmeier M, Dura JM, Paro R. 2003. Genome-wide prediction of Polycomb/Trithorax response elements in Drosophila melanogaster. *Dev Cell* **5**: 759-771.

Sandmann T, Jakobsen JS, Furlong EE. 2006. ChIP-on-chip protocol for genome-wide analysis of transcription factor binding in Drosophila melanogaster embryos. *Nat Protoc* **1**: 2839-2855.

Schuettengruber B, Ganapathi M, Leblanc B, Portoso M, Jaschek R, Tolhuis B, van Lohuizen M, Tanay A, Cavalli G. 2009. Functional anatomy of polycomb and trithorax chromatin landscapes in Drosophila embryos. *PLoS Biol* **7**: e13.

Schuettengruber B, Oded Elkayam N, Sexton T, Entrevan M, Stern S, Thomas A, Yaffe E, Parrinello H, Tanay A, Cavalli G. 2014. Cooperativity, specificity, and evolutionary stability of Polycomb targeting in Drosophila. *Cell Rep* **9**: 219-233.

Shimell MJ, Peterson AJ, Burr J, Simon JA, O'Connor MB. 2000. Functional analysis of repressor binding sites in the iab-2 regulatory region of the abdominal-A homeotic gene. *Dev Biol* **218**: 38-52.

St Pierre SE, Ponting L, Stefancsik R, McQuilton P. 2014. FlyBase 102--advanced approaches to interrogating FlyBase. *Nucleic Acids Res* **42**: D780-788.

Von Ohlen T, Hooper JE. 1997. Hedgehog signaling regulates transcription through Gli/Ci binding sites in the wingless enhancer. *Mech Dev* **68**: 149-156.

Weiss A, Charbonnier E, Ellertsdottir E, Tsirigos A, Wolf C, Schuh R, Pyrowolakis G, Affolter M. 2010. A conserved activation element in BMP signaling during Drosophila development. *Nat Struct Mol Biol* **17**: 69-76.

Zinzen RP, Girardot C, Gagneur J, Braun M, Furlong EE. 2009. Combinatorial binding predicts spatio-temporal cis-regulatory activity. *Nature* **462**: 65-70.
